# Application Potential of Baijiu Non-*Saccharomyces* Yeast in Winemaking Through Sequential Fermentation With *Saccharomyces cerevisiae*

**DOI:** 10.3389/fmicb.2022.902597

**Published:** 2022-05-30

**Authors:** Rui-Rui Li, Meng Xu, Jia Zheng, Yan-Jun Liu, Chun-Hong Sun, Huan Wang, Xue-Wu Guo, Dong-Guang Xiao, Xiao-Le Wu, Ye-Fu Chen

**Affiliations:** ^1^Key Laboratory of Industrial Fermentation Microbiology, Ministry of Education, Tianjin Industrial Microbiology Key Laboratory, College of Biotechnology, Tianjin University of Science and Technology, Tianjin, China; ^2^Key Laboratory of Wuliangye-Flavor Liquor Solid-State Fermentation, China National Light Industry, Yibin, China

**Keywords:** *S. cerevisiae*, *Zygosaccharomyces bailii*, *Pichia kudriavzevii*, sequential fermentation, volatile compounds

## Abstract

To explore the potential application of non-*Saccharomyces* yeasts screened from Baijiu fermentation environment in winemaking, the effect of four Baijiu non-*Saccharomyces* yeasts (two *Zygosaccharomyces bailii* and two *Pichia kudriavzevii*) sequentially fermented with *Saccharomyces cerevisiae* on the physicochemical parameters and volatile compounds of wine was analyzed. The results indicated that there was no obvious antagonism between *S. cerevisiae* and *Z. bailli* or *P. kudriavzevii* in sequential fermentations, and all strains could be detected at the end of alcoholic fermentation. Compare with *S. cerevisiae* pure fermentation, *Z. bailii/S. cerevisiae* sequential fermentations significantly reduced higher alcohols, fatty acids, and ethyl esters and increased acetate esters; *P. kudriavzevii/S. cerevisiae* sequential fermentations reduced the contents of C6 alcohols, total higher alcohols, fatty acids, and ethyl esters and significantly increased the contents of acetate esters (especially ethyl acetate and 3-methylbutyl acetate). Sequential fermentation of Baijiu non-*Saccharomyces* yeast and *S. cerevisiae* improved the flavor and quality of wine due to the higher ester content and lower concentration of higher alcohols and fatty acids, non-*Saccharomyces* yeasts selected from Baijiu fermentation environment have potential applications in winemaking, which could provide a new strategy to improve wine flavor and quality.

## Introduction

Studies have reported that some non-*Saccharomyces* yeasts can improve the organoleptic quality and sensory notes of wine, depending on the specific yeast species and strains used (Padilla et al., 2016; Benito et al., 2019). For example, most species from the *Hanseniospora* genus can improve the color and polyphenolic composition in red wines (Leixà et al., 2016), *Torulaspora delbrueckii* and *Saccharomyces cerevisiae* sequential inoculation can increase total esters concentration such as 3-methylbutyl acetate, ethyl propanoate, and ethyl 2-methylpropanoate (Renault et al., 2015), *Pichia anomala* and *S. cerevisiae* mixed fermentation can increase the content of 3-methylbutyl acetate and ethyl esters (Kurita, 2008). However non-*Saccharomyces* yeasts selected from vineyards (including the grapes) and wineries (including the winery equipment) have a low capacity to metabolize sugar to ethanol, and low resistance to sulfur dioxide and ethanol in most cases, they have often been inhibited by *S. cerevisiae* inoculations at the industrial level (Benito, 2018). Therefore, it is necessary to look for excellent non-*Saccharomyces* yeasts with high ability to ferment sugar, high tolerance to various stresses, and can produce high yield aroma compounds to be used in winemaking.

Baijiu, a traditional fermented alcoholic beverage in China, is rich in many flavor components, including esters, terpenes, organic acids, lactones, phenols, heterocycles, and aromatic compounds (Fan and Qian, 2006). Baijiu fermentation is generally under an open or semi-open fermentation environment (Xu et al., 2022), a variety of microorganisms from the Daqu (the fermentation starter), water, air, tools, and operators participate in the fermentation process (Xu et al., 2017a). Among these fermentation microbial communities, non-*Saccharomyces* yeasts can produce aldehydes, esters, higher alcohols, and other flavor substances during Baijiu fermentation, giving Baijiu its typical aroma characteristics. For example, *Zygosaccharomyces bailii* is a type of yeast with high tolerance to various stresses (Stratford et al., 2013; Palma et al., 2015). It was found to be a dominant species in Maotai-flavor liquor fermentation and the contributors to ethanol and various flavor compounds in Baijiu making (Xu et al., 2017b). In addition, it was found that the *Zygosaccharomyces* strains isolated from grape musts were described as strains with low higher alcohol production (Romano and Suzzi, 1993). In wine fermentation, the high ester-producing ability of *Z. bailii* was used to increase the content of ethyl ester in wine by mixed fermentation with *S. cerevisiae* (Ciani et al., 2010; Garavaglia et al., 2015). *Pichia kudriavzevii* is the dominant species of the genus *Pichia* in Baijiu fermentation (Jiang et al., 2019), which can generate esters, higher alcohols, and volatile acids (Liu et al., 2017). In addition, *P. kudriavzevii* can produce higher glycerol, ethyl acetate, and 3-methylbutyl acetate in mixed fermentation of wine but lower contents of fatty acids, higher alcohols, and phenylethyl alcohol (Luan et al., 2018). Shi et al. (2019) found that the wine fermented by *P. kudriavzevii* and *S. cerevisiae* had lower concentrations of volatile acids, higher alcohols, fatty acids, benzene derivatives, and C6 compounds, and higher concentrations of esters, which improved the aroma and overall flavor characteristics of the wine.

The production process of Baijiu is accompanied by special extreme environments, such as high ethanol, high temperature, and high acidity (Xu et al., 2017a; Wang et al., 2019). After long-term domestication in an extreme environment, Baijiu non-*Saccharomyces* yeast strains may have stronger ability to adapt to the winemaking environment. The non-*Saccharomyces* yeast strains selected from Baijiu may not be easily inhibited by *S. cerevisiae* in wine fermentation, which is more beneficial to improving the quality of wine. Therefore, the co-fermentation of non*-Saccharomyces* yeast strains selected from Baijiu fermentation environments and *S. cerevisiae* may be a new strategy to improve wine flavor and quality. To the best of our knowledge, one non-*Saccharomyces* yeast strain (*Pichia fermentans*) selected from Baijiu fermentation environments has been applied to wine fermentation, and it showed a positive contribution to wine aroma (Ma et al., 2017; Li et al., 2020).

In the previous work at our laboratory, several non-*Saccharomyces* yeasts were isolated from the fermented grains of a sauce-flavor Baijiu in China. Previous studies demonstrated that they have good fermentation characteristics and show high ester-producing ability (unpublished results). To explore the application potential of Baijiu non-*Saccharomyces* yeast strains in wine fermentation, in this study, four non-*Saccharomyces* yeast strains (two *Z. bailii* and two *P. kudriavzevii*) selected from the fermented grains of a sauce-flavor Baijiu in China were inoculated sequentially with *S. cerevisiae* EC1118, and their effects on the physicochemical parameters and volatile compositions of wine were evaluated.

## Materials and Methods

### Yeast Strains and Preculture Conditions

Four indigenous non-*Saccharomyces* yeast strains were used in this study, including *Z. bailii* (BJII45005 and BJVI11007) and *P. kudriavzevii* (BJIV53006 and BJII44006). The four strains were isolated from the fermented grains of a sauce-flavor Baijiu in China. They were identified by 26S rDNA analysis (Supplementary Table 1) and kept in our lab. The commercial wine strain *S. cerevisiae* Lalvin EC1118 (Lallemand Inc., Montreal, QC, Canada) was used as the control. These strains were stored at –80^°^C in YPD medium with glycerol (20% v/v final concentration).

Starter cultures of all yeast strains were prepared by inoculating a single colony in 5 mL of YPD medium broth for each strain. The cultures were incubated in a test tube rotating overnight (30^°^C and 180 rpm). These starter cultures were transferred to 500-mL shake flasks containing 300 mL of YPD medium for 18 h (30^°^C and 180 rpm). The commercial strain EC1118 was prepared under the same conditions as non-*Saccharomyces* yeast. The cultured yeast strains were counted by the blood cell counting method, and each sample was counted in triplicate. The yeast cells were centrifuged and washed twice with sterile water. The inoculum ratio of non-*Saccharomyces* yeast and *S. cerevisiae* was 10:1, and the initial active population of non-*Saccharomyces* yeast and *S. cerevisiae* was 1.0 × 10^7^ cells/mL and 1.0 × 10^6^ cells/mL (Zhang et al., 2018), respectively.

### Fermentation Conditions and Sampling

*Cabernet Sauvignon* grapes (227.54 g/L of sugar, pH 3.38, 259.81 mg/L yeast assimilable nitrogen) were harvested from the Qinhuangdao region vineyard (Hebei, China) in the 2020 vintage (October 3rd). The grapes were in good sanitary conditions and came from the same vineyard. The grapes were harvested by hand and immediately transported to the laboratory in the same box. After stemming and crushing, 400 g of grape musts were added into 500-mL Erlenmeyer flasks and pasteurized at 68^°^C for 30 min. The grape musts were macerated at 4^°^C for 12 h after 60 mg/L total SO_2_ was added. When the temperature returned to 25^°^C, yeast was added. Flasks were sealed with hydrophobic membranes to create anaerobic conditions (carbon dioxide was released through an air outlet membrane).

Mixed fermentation trials were performed with four indigenous non-*Saccharomyces* yeasts and EC1118 sequential inoculation. Nine trials were therefore set: (1) single inoculation with EC1118 (SC); (2) sequential inoculation with BJVI11007, followed by inoculation with EC1118 after 2 days (ZBI-2); (3) sequential inoculation with BJVI11007, followed by inoculation with EC1118 after 3 days (ZBI-3); (4) sequential inoculation with BJII45005, followed by inoculation with EC1118 after 2 days (ZBII-2); (5) sequential inoculation with BJII45005, followed by inoculation with EC1118 after 3 days (ZBII-3); (6) sequential inoculation with BJIV53006, followed by inoculation with EC1118 after 2 days (PKI-2); (7) sequential inoculation with BJIV53006, followed by inoculation with EC1118 after 3 days (PKI-3); (8) sequential inoculation with BJII44006, followed by inoculation with EC1118 after 2 days (PKII-2); and (9) sequential inoculation with BJII44006, followed by inoculation with EC1118 after 3 days (PKII-3). Fermentations were carried out in triplicate for each treatment at a controlled temperature of 25^°^C and included punching skins down twice a day to improve extraction. A total of 2 mL of fermenting musts were sampled daily for counting the yeast population and for HPLC analysis.

### The Fermentation Process and Yeast Enumeration

The fermentation process was monitored twice per day by measuring the weight loss of the bottles due to the CO_2_ release. The fermentations were stopped when the weight loss was less than 0.1 g in 12 h. After alcoholic fermentation, grape pomace was separated from wine carefully, centrifuged, and stored at –20^°^C for further analysis.

The viable cell count was performed by identifying colony colors using 100 mg/L of chloramphenicol (Solarbio, Beijing, China) that was added to Wallerstein Laboratory (WLN) nutrient agar (Qingdao Hope Bio-Technology Co., Ltd., China). One hundred microliter aliquots were plated onto WLN plates. After 48 h of incubation at 30^°^C, the cells could be differentially counted based on the morphological particularities presented by the non-*Saccharomyces* yeasts that distinguished them from *S. cerevisiae*.

### Analysis of Physicochemical Parameters

Ethanol, glucose, fructose, glycerol, citric acid, tartaric acid, malic acid, succinic acid, lactic acid, and acetic acid were determined by HPLC (Agilent 1260 Infinity) system consisting of a quaternary gradient pump (1260 Iso Pump-G1310B), an autosampler (1260 ALS-C1328B) and a refractive index detector (1260 TCC-G1316A). Separations were performed on a Silgreen Ca/H column operating at 65^°^C, the mobile phase was 5 mM sulfuric acid at a flow rate of 0.6 mL min^–1^, and the running time of the program was 23 min. The samples were diluted and filtered (0.22 μm RC syringe filters, Tianjin Jinteng Experimental Equipment Equipment Co., Ltd., Tianjin, China) and 20 μL were injected. The concentration of each metabolite was calculated with an external standard method using peak areas. The detailed quantitation information about quantitative standards, calibration curves, and *R*^2^ for the quantification compounds used in this study was provided in Supplementary Table 2. The pH of the wine samples was measured using a pHSJ-4A model pH meter (Shanghai Scientific Instruments and Materials Co., Ltd.).

### Analysis of Volatile Compounds

The volatile compounds of wines after alcohol fermentation were quantified using headspace solid-phase microextraction coupled with gas chromatography-mass spectrometry (HS-SPME-GC-MS) according to our previous study (Li and Sun, 2019). Identification was based on ion fragment and mass spectra matching against the standard NIST 14 library. 4-Methyl-2-pentanol was used as the internal standard (20 μL of 2.00 g/L sample solution). Subsequently, all standard stock solutions were combined, and this mixed standard solution was diluted into several levels in succession with a 12% (v/v) alcohol solution. Under the same conditions, the standards at all levels were extracted and analyzed. For quantification, calibration curves were obtained with regression coefficients above 98% (Supplementary Table 3). In addition, the concentration of volatile compounds without pure standard was estimated based on the calibration curves of standard compounds with the most similar chemical structures and/or numbers of carbon atoms (Cai et al., 2014).

### Sensory Evaluation

The sensory evaluation was performed as described by Zhang et al. (2022) with modification. Twenty milliliters of wine samples were poured into wine glasses and presented in random order. Wine sensory evaluation was classified into nine attributes, including aromatic intensity, floral, fruity, sweet, herbaceous, fatty, solvent, acidity, and wine body. The descriptive sensory analysis was carried out by a well-trained sensory panel comprised of 12 assessors from the College of Biotechnology, Tianjin University of Science and Technology (six females and six males, ranging in ages from 22 to 36, an average of 25). Panelists were required to rate the intensity of the wine parameters using a 9-point scale (1 = extremely low, 5 = moderate intensity, 9 = extremely high). The final score of each sensory characteristic was the mean value of scores given by 12 assessors.

### Statistical Analysis

One way analysis of variance (ANOVA) using the Duncan test at the significance level of *P* < 0.05 was performed using SPSS 17.0 (Chicago, IL, United States), Principal component analysis (PCA) was conducted using the SIMCA-13.0 software (Umetrics, Sweden); Heat map was conducted using the TBtools software (Chen et al., 2020); Upset plot was conducted using the R software (version 3.3.2) in UpSetR package; others were conducted using the OriginPro81 (OriginLab, United States).

## Results

### The Fermentation Process and Yeast Growth During Fermentation

**FIGURE 1:**
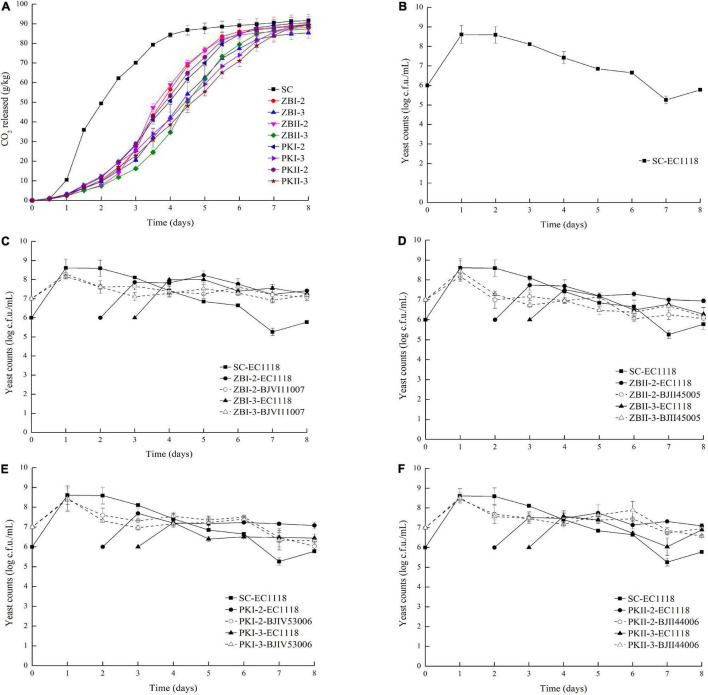
CO_2_ released and yeast population during alcoholic fermentation. **(A)** CO_2_ released during alcoholic fermentation; **(B)** yeast population of *S. cerevisiae* EC1118 pure fermentation; **(C)** yeast population of *Z. bailii* BJVI11007/*S. cerevisiae* EC1118 co-fermentation; **(D)** yeast population of *Z. bailii* BJII45005/*S. cerevisiae* EC1118 co-fermentation; **(E)** yeast population of *P. kudriavzevii* BJIV53006/*S. cerevisiae* EC1118 co-fermentation; **(F)** yeast population of *P. kudriavzevii* BJII44006/*S. cerevisiae* EC1118 co-fermentation.

The fermentation process (represented by CO_2_ release) and yeast growth kinetics in pure fermentation and sequential fermentations are shown in Figure 1. Alcohol fermentation lasted for 8 days, and no stuck or sluggish fermentations were found in any of the inoculation strategies. *S. cerevisiae* EC1118 single inoculation finished alcohol fermentation faster, showing that sequential inoculations slowed down the end of the process. Compared with *S. cerevisiae* pure fermentation, sequential fermentation delayed the time of alcohol fermentation (Figure 1A). In sequential fermentations, the inoculation time of *S. cerevisiae* had a great effect on the fermentation alcohol process, and the delayed inoculation for 2 days was faster than that for 3 days. Similarly, SC consumed glucose and fructose faster than sequential fermentations (Supplementary Figures 1A,B).

The influence of yeast strains on the final flavor characteristics of the wine is largely determined by the yeast cell number during the fermentation process (Zhang et al., 2018). In *S. cerevisiae* pure fermentation (Figure 1B), EC1118 grew rapidly on the first day of inoculation before reaching a maximum (4.07 × 10^8^ CFU/mL) and then gradually decreased until the end of alcohol fermentation. In sequential fermentations (Figures 1C–F), two non-*Saccharomyces* yeasts of the same species showed a similar growth curve. In the sequential fermentation of *Z. bailli* and *S. cerevisiae* (ZB) (Figures 1C,D), the population of *Z. bailli* increased slightly on the first day of inoculation and then decreased gradually in the subsequent fermentation process. The number of EC1118 increased rapidly after inoculation, and the population of EC1118 was higher than that of *Z. bailli* in the subsequent fermentation process. In the sequential fermentation of *P. kudriavzevii* and *S. cerevisiae* (PK) (Figures 1E,F), *P. kudriavzevii* grew rapidly on the first day and then slightly decreased, and the quantity was maintained in the first 6 days. Compared with the inoculation of *S. cerevisiae* on the second day, the number of *S. cerevisiae* inoculated on the third day was lower than that of *P. kudriavzevii*. In summary, sequential fermentation delayed the time of alcohol fermentation, and no obvious antagonism between *S. cerevisiae* and *Z. bailli* or *P. kudriavzevii*, and all strains could be detected at the end of alcohol fermentation.

### Physicochemical Parameters

The physicochemical parameters of wines produced by pure and sequential fermentations are shown in Table 1. At the end of alcohol fermentation, all treatments were completely fermented (residual sugars were less than 2 g/L). There was no significant difference in the content of ethanol, and no regular difference in the pH value among the samples. In this study, the acetic acid content in all wine samples was below 0.80 g/L, and PK could significantly decrease the acetic acid content. In terms of non-volatile acid, the citric acid content in ZB was significantly lower than that in SC, and the effects of *P. kudriavzevii* were slightly different between the two strains. PK could significantly increase the content of tartaric acid. Compared with SC, sequential fermentations could significantly increase the content of malic acid and reduce the content of lactic acid. It was worth noting that ZB could significantly increase the content of succinic acid.

**TABLE 1:** Physicochemical parameters (g/L) of the final wines after alcoholic fermentation.

Compounds	SC	ZBI-2	ZBI-3	ZBII-2	ZBII-3	PKI-2	PKI-3	PKII-2	PKII-3
Ethanol	92.14 ± 5.52a	90.41 ± 2.00a	88.82 ± 2.33a	89.83 ± 2.03a	90.13 ± 1.51a	92.95 ± 2.07a	91.25 ± 2.72a	91.86 ± 1.77a	91.77 ± 3.01a
Glycerol	11.14 ± 0.09cd	11.58 ± 0.04ab	11.74 ± 0.03a	11.66 ± 0.59ab	10.89 ± 0.16d	11.22 ± 0.23bcd	11.14 ± 0.04cd	11.05 ± 0.19cd	11.40 ± 0.19abc
Glucose	0.21 ± 0.04f	0.87 ± 0.04b	0.99 ± 0.07a	0.51 ± 0.04d	0.22 ± 0.07f	0.36 ± 0.08e	0.80 ± 0.02b	0.68 ± 0.17c	0.33 ± 0.15e
Fructose	0.27 ± 0.06c	0.51 ± 0.10b	0.33 ± 0.17bc	1.05 ± 0.13a	1.09 ± 0.17a	1.17 ± 0.32a	1.05 ± 0.18a	0.30 ± 0.07c	1.16 ± 0.13a
pH	3.63 ± 0.05bc	3.72 ± 0.08ab	3.56 ± 0.02c	3.68 ± 0.06b	3.65 ± 0.01b	3.77 ± 0.06a	3.71 ± 0.03ab	3.65 ± 0.04b	3.65 ± 0.02b
Citric acid	1.61 ± 0.01c	1.10 ± 0.01e	1.29 ± 0.09d	1.19 ± 0.06de	0.76 ± 0.02f	1.80 ± 0.07b	1.95 ± 0.12a	1.27 ± 0.08d	1.08 ± 0.04e
Tartaric acid	2.13 ± 0.08cd	2.08 ± 0.08d	2.14 ± 0.05cd	2.21 ± 0.09bc	2.02 ± 0.01d	2.35 ± 0.09a	2.39 ± 0.08a	2.27 ± 0.03ab	2.35 ± 0.06a
Malic acid	1.88 ± 0.08f	1.93 ± 0.04ef	3.16 ± 0.10a	2.12 ± 0.08d	2.77 ± 0.12b	1.98 ± 0.05ef	2.32 ± 0.10c	2.04 ± 0.03de	2.14 ± 0.01d
Succinic acid	2.02 ± 0.03d	3.62 ± 0.08b	3.88 ± 0.10a	3.51 ± 0.14b	3.36 ± 0.04c	1.30 ± 0.01e	1.35 ± 0.03e	1.38 ± 0.03e	1.37 ± 0.04e
Lactic acid	0.42 ± 0.01a	0.18 ± 0.03e	0.14 ± 0.01f	0.25 ± 0.02b	0.17 ± 0.01e	0.21 ± 0.01cd	0.23 ± 0.01bc	0.16 ± 0.01ef	0.19 ± 0.01de
Acetic acid	0.40 ± 0.03c	0.61 ± 0.11b	0.73 ± 0.07a	0.30 ± 0.05d	0.26 ± 0.02d	0.26 ± 0.02d	0.26 ± 0.01d	0.24 ± 0.02d	0.25 ± 0.01d

*SC, single inoculation with S. cerevisiae EC1118; ZBI-2, sequential inoculation with Z. bailii BJVI11007, followed by inoculation with EC1118 after 2 days; ZBI-3, sequential inoculation with Z. bailii BJVI11007, followed by inoculation with EC1118 after 3 days; ZBII-2, sequential inoculation with Z. bailii BJII45005, followed by inoculation with EC1118 after 2 days; ZBII-3, sequential inoculation with Z. bailii BJII45005, followed by inoculation with EC1118 after 3 days; PKI-2, sequential inoculation with P. kudriavzevii BJIV53006, followed by inoculation with EC1118 after 2 days; PKI-3, sequential inoculation with P. kudriavzevii BJIV53006, followed by inoculation with EC1118 after 3 days; PKII-2, sequential inoculation with P. kudriavzevii BJII44006, followed by inoculation with EC1118 after 2 days; PKII-3, sequential inoculation with P. kudriavzevii BJII44006, followed by inoculation with EC1118 after 3 days. Values are given as mean ± standard deviation of three replicates. Data with different letters (a, b, c, d, e, f, g, h) within each row are different according to Duncan tests (p ≤ 0.05).*

### Volatile Compositions

**FIGURE 2:**
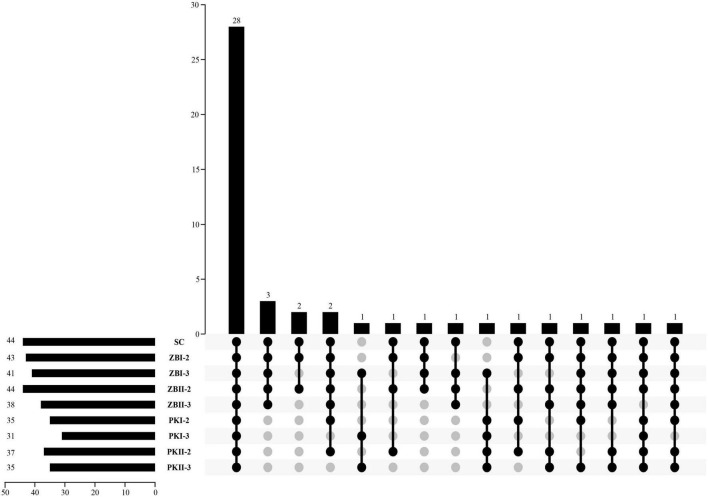
The distributions of wine volatile components in different fermentation trials. The bar chart at the bottom left represents the kinds of volatile components included in each wine sample. The bar chart above represents the kinds of common volatile compounds in the wine samples. The black dot connected by the solid line at the bottom right shows the wine samples contained in the group, and the gray dot shows the wine sample is not included in the group.

The volatile compositions of the wines produced by different inoculation strategies were determined. Forty-six volatile compositions were identified in all samples, including 17 alcohols (three C6 alcohols and 14 higher alcohols), three fatty acids, 22 esters (four acetate esters, 13 ethyl esters, and five other esters), and four other compounds (Table 2). Compared with SC, PK increased the total content of volatile compositions, especially BJII44006. ZB could significantly reduce the total content of volatile compositions. The kinds of volatile compounds affect the complexity of the wine aroma, the Upset plot (Figure 2) was used to visualize the difference in the kinds of volatile compounds among different inoculation strategies. Among them, 28 kinds of volatile compositions were detected in all samples (Figure 2); SC had the most kinds (44 kinds) of volatile compositions, followed by ZBI-2 (43 kinds), and PKI-3 (31 kinds) had the least kinds of volatile compositions. In general, compared with SC, sequential fermentations could reduce the kinds of volatile compositions; the inoculation of *S. cerevisiae*, which was delayed by 3 days, reconfirmed this result. The odor activity value (OAV) was calculated as the ratio between the concentration of each volatile compound and its perception threshold. The compound that OAV over one has a high contribution to wine aroma (Guth, 1997). In recent years, some researchers (Escudero et al., 2007; Ryan et al., 2008) found that compounds with relatively low OAVs can have an unexpectedly high effect on the aroma. So, the ratios of volatile compound contents (OAV > 0.1) after alcoholic fermentation were calculated and are shown in Supplementary Table 4.

**TABLE 2:** Volatile composition (μg/L) of the final wines after alcoholic fermentation.

Aroma compounds	SC	ZBI-2	ZBI-3	ZBII-2	ZBII-3	PKI-2	PKI-3	PKII-2	PKII-3
1-Hexanol	629.71 ± 19.85ab	611.50 ± 46.49ab	738.30 ± 34.61a	735.14 ± 129.86a	738.55 ± 95.45a	407.66 ± 23.63d	574.76 ± 56.58bc	604.32 ± 75.30b	470.00 ± 55.43cd
(E)-3-Hexen-1-ol	182.08 ± 25.13b	193.76 ± 2.65ab	195.88 ± 2.07ab	205.66 ± 2.82a	202.13 ± 5.77a	200.28 ± 3.09a	194.64 ± 3.61ab	210.47 ± 5.77a	196.98 ± 1.39ab
(Z)-3-Hexen-1-ol	196.51 ± 7.41abc	187.28 ± 5.51cd	200.03 ± 3.87ab	201.34 ± 10.94a	193.23 ± 3.20abcd	184.54 ± 3.95d	189.89 ± 2.71abc	192.04 ± 1.60abcd	187.41 ± 2.04cd
**Total C6 alcohols**	**1008.30 ± 50.41ab**	**992.54 ± 53.05b**	**1134.22 ± 32.38a**	**1142.14 ± 136.84a**	**1133.91 ± 103.31a**	**792.48 ± 27.96d**	**959.30 ± 60.92bc**	**1006.82 ± 76.54ab**	**854.38 ± 58.75cd**
1-Butanol	4040.30 ± 345.55a	772.79 ± 37.30c	124.96 ± 8.20e	1329.87 ± 241.77b	409.16 ± 43.42d	441.79 ± 36.29d	362.64 ± 38.73de	581.47 ± 57.34cd	564.28 ± 46.96cd
2-Methyl-1-propanol (mg/L)	47.59 ± 2.20f	54.95 ± 3.62ef	80.88 ± 8.33d	64.47 ± 5.81e	66.86 ± 1.49e	145.93 ± 11.17ab	152.29 ± 13.66a	123.62 ± 10.36c	133.97 ± 6.64bc
3-Methyl-1-butanol (mg/L)	270.47 ± 9.58a	161.82 ± 1.70e	170.89 ± 3.68de	230.98 ± 26.34b	154.01 ± 5.77e	195.54 ± 11.70c	185.78 ± 6.02cd	220.04 ± 10.95b	215.61 ± 5.02b
3-Methyl-1-pentanol	506.55 ± 24.01a	240.49 ± 18.72c	209.25 ± 4.01d	312.51 ± 29.96b	223.79 ± 6.12cd	207.11 ± 1.35d	204.71 ± 1.73d	216.63 ± 2.10cd	214.85 ± 0.93cd
4-Methyl-1-pentanol	248.78 ± 1.97a	202.89 ± 3.45c	Nd	217.59 ± 5.42b	199.11 ± 0.89c	Nd	Nd	202.63 ± 0.21c	201.92 ± 1.09c
1-Heptanol	115.14 ± 20.22a	16.20 ± 0.58c	Nd	30.05 ± 8.22b	Nd	Nd	Nd	Nd	Nd
1-Octanol	4.90 ± 0.24a	0.80 ± 0.19c	Nd	2.09 ± 0.09b	Nd	Nd	Nd	Nd	Nd
1-Non-anol	Nd	Nd	18.61 ± 0.01a	Nd	Nd	Nd	18.00 ± 0.12b	Nd	16.51 ± 0.03c
2-Non-anol	2.90 ± 0.01d	3.77 ± 0.47c	4.16 ± 0.37b	4.40 ± 0.14b	5.56 ± 0.16a	3.14 ± 0.02d	Nd	3.11 ± 0.01d	Nd
1-Decanol	18.15 ± 0.23a	16.88 ± 0.11c	16.33 ± 0.30de	17.54 ± 0.65b	16.51 ± 0.07cd	15.88 ± 0.19e	Nd	16.10 ± 0.09de	Nd
Benzyl alcohol	171.68 ± 19.13a	88.70 ± 7.45d	90.69 ± 5.37cd	117.96 ± 15.45b	108.60 ± 0.63bc	98.38 ± 16.30cd	27.59 ± 2.67e	83.01 ± 6.64d	12.32 ± 2.68e
Phenylethyl alcohol (mg/L)	248.34 ± 7.74a	57.14 ± 6.52cd	40.10 ± 4.42d	157.63 ± 28.75b	51.84 ± 7.65cd	53.12 ± 4.73cd	50.63 ± 2.62cd	66.87 ± 3.52c	59.91 ± 4.55cd
*Leavo*-2,3-Butanediol (mg/L)	44.51 ± 1.23a	25.48 ± 2.91cd	33.84 ± 6.05b	35.91 ± 6.26b	31.02 ± 3.02bc	44.36 ± 0.93a	23.54 ± 1.13d	26.53 ± 1.39cd	25.13 ± 2.07cd
*Meso*-2,3-Butanediol (mg/L)	15.91 ± 0.94d	7.80 ± 0.92e	13.54 ± 1.02d	8.64 ± 0.71e	9.88 ± 0.76e	26.02 ± 2.77c	27.52 ± 1.50bc	30.22 ± 2.13ab	31.36 ± 3.13a
**Total higher alcohols (mg/L)**	**631.93 ± 4.59a**	**308.54 ± 5.83d**	**339.71 ± 2.67d**	**499.66 ± 67.47b**	**314.57 ± 16.83d**	**465.72 ± 23.78bc**	**440.37 ± 20.59c**	**468.38 ± 24.88bc**	**467.00 ± 8.76bc**
Hexanoic acid	739.90 ± 72.57a	184.39 ± 15.85bc	117.29 ± 1.53d	233.68 ± 60.55b	138.13 ± 1.00cd	Nd	Nd	Nd	Nd
Octanoic acid	366.86 ± 48.47a	49.60 ± 5.31c	16.13 ± 2.00d	98.97 ± 9.07b	47.21 ± 7.77c	Nd	Nd	25.60 ± 6.17cd	12.58 ± 1.00d
Decanoic acid	8.65 ± 0.50b	13.03 ± 0.02a	8.24 ± 0.15c	8.24 ± 0.12c	8.30 ± 0.04c	Nd	Nd	Nd	Nd
**Total fatty acids**	**1115.41 ± 85.16a**	**247.03 ± 20.90c**	**141.66 ± 1.20d**	**340.88 ± 68.32b**	**193.64 ± 8.39cd**	**Nd**	**Nd**	**25.60 ± 6.17e**	**12.58 ± 1.00e**
Ethyl acetate (mg/L)	46.96 ± 2.26h	67.27 ± 6.11fg	155.85 ± 17.39e	48.87 ± 8.18gh	72.29 ± 2.11f	248.01 ± 10.57d	270.84 ± 16.48c	318.55 ± 15.36b	369.25 ± 8.98a
3-Methylbutyl acetate	294.91 ± 30.54d	324.97 ± 57.40d	364.40 ± 81.11d	274.68 ± 9.30d	304.95 ± 19.12d	1181.00 ± 76.21c	1473.47 ± 295.29b	1540.21 ± 289.05b	2160.99 ± 265.52a
Phenethyl acetate	154.45 ± 16.87abc	110.91 ± 7.54f	119.07 ± 7.66f	145.19 ± 11.82bcd	123.82 ± 6.14 ef	128.46 ± 6.57def	138.14 ± 9.24cde	162.23 ± 10.04ab	169.34 ± 11.37a
2-Methylpropyl acetate	60.01 ± 12.29d	78.67 ± 6.67cd	90.02 ± 6.45c	58.89 ± 11.64d	69.73 ± 7.78cd	248.05 ± 17.27b	267.54 ± 6.62b	246.62 ± 30.37b	311.34 ± 16.62a
**Total acetate esters (mg/L)**	**47.47 ± 2.24h**	**67.79 ± 6.17fg**	**156.42 ± 17.36e**	**49.35 ± 8.17gh**	**72.78 ± 2.14f**	**249.57 ± 10.50d**	**272.72 ± 16.78c**	**320.50 ± 15.07b**	**371.89 ± 8.98a**
Ethyl propanoate	224.67 ± 27.15e	274.57 ± 24.17de	557.89 ± 32.82c	328.29 ± 19.76d	312.35 ± 15.21d	684.09 ± 32.09a	710.11 ± 51.24a	619.23 ± 24.19b	686.04 ± 36.38a
Ethyl butanoate	173.29 ± 11.29ab	197.58 ± 9.60a	184.81 ± 16.06ab	167.82 ± 28.91b	192.84 ± 17.40ab	139.24 ± 8.88c	113.80 ± 0.44d	106.07 ± 6.61d	103.37 ± 1.42d
Ethyl 2-methylpropanoate	39.10 ± 1.20f	42.64 ± 1.12f	58.02 ± 5.23e	41.40 ± 2.15f	43.42 ± 1.21f	133.53 ± 12.51d	149.09 ± 2.87c	161.57 ± 14.62b	200.12 ± 5.64a
Ethyl hexanoate	369.72 ± 26.62a	145.28 ± 17.59b	41.68 ± 8.30d	160.70 ± 22.63b	77.96 ± 12.59c	73.45 ± 9.03c	61.74 ± 9.29cd	84.15 ± 10.06c	39.80 ± 8.03d
Ethyl 2-hexenoate	Nd	Nd	6.31 ± 0.06b	Nd	Nd	7.97 ± 0.73a	8.41 ± 1.21a	8.24 ± 0.84a	7.42 ± 0.50a
Ethyl heptanoate	6.87 ± 0.75a	3.69 ± 0.15c	4.09 ± 0.96bc	4.04 ± 0.36bc	3.57 ± 0.35c	4.97 ± 0.71b	4.19 ± 0.28bc	4.54 ± 0.20bc	3.84 ± 0.17c
Ethyl octanoate	748.83 ± 70.80a	204.24 ± 25.37b	63.74 ± 7.33e	219.55 ± 75.42b	122.01 ± 9.26cde	162.39 ± 58.66bc	74.11 ± 9.14de	146.79 ± 9.75bcd	73.63 ± 8.30de
Ethyl decanoate	567.55 ± 66.16a	107.02 ± 12.05c	33.20 ± 3.18d	173.12 ± 22.76b	74.29 ± 11.92cd	56.32 ± 3.63d	33.00 ± 0.71d	62.37 ± 7.98d	34.96 ± 2.33d
Ethyl phenylacetate	50.17 ± 1.13a	46.65 ± 1.00de	46.20 ± 0.27e	49.28 ± 1.26ab	Nd	48.06 ± 0.23bc	47.65 ± 0.13cd	48.80 ± 0.25bc	49.17 ± 0.30ab
Ethyl undecanoate	34.54 ± 0.02a	33.95 ± 0.58b	Nd	34.40 ± 0.12a	Nd	Nd	Nd	34.28 ± 0.06ab	Nd
Ethyl dodecanoate	138.47 ± 16.41a	43.62 ± 1.54bc	38.63 ± 4.15c	52.26 ± 8.44b	40.39 ± 1.82c	37.77 ± 0.77c	35.97 ± 0.08c	37.90 ± 0.73c	36.11 ± 0.31c
Ethyl tetradecanoate	36.96 ± 0.28a	34.24 ± 1.00b	34.27 ± 0.01b	34.08 ± 0.91b	Nd	Nd	Nd	Nd	Nd
Diethyl succinate	22.32 ± 2.40a	15.73 ± 0.68bc	10.54 ± 2.39d	16.63 ± 1.87b	13.64 ± 1.57c	Nd	Nd	Nd	Nd
**Total ethyl esters**	**2412.48 ± 212.00a**	**1149.19 ± 27.95cd**	**1079.40 ± 63.74d**	**1270.48 ± 165.29bc**	**880.47 ± 37.57e**	**1347.80 ± 32.03b**	**1238.07 ± 64.83bcd**	**1313.95 ± 34.76bc**	**1234.47 ± 56.66bcd**
Methyl decanoate	54.25 ± 0.33a	Nd	53.51 ± 0.01b	53.54 ± 0.07b	53.50 ± 0.06b	Nd	Nd	Nd	Nd
2-Methylpropyl decanoate	34.95 ± 0.09a	34.18 ± 0.02b	Nd	34.37 ± 0.02ab	Nd	33.86 ± 0.58b	Nd	34.26 ± 0.75b	Nd
Octanoic acid, 3-methylbutyl ester	54.83 ± 1.46a	36.47 ± 0.44c	36.05 ± 0.01cd	38.52 ± 1.69b	35.68 ± 0.32cd	35.24 ± 0.29cd	Nd	35.62 ± 0.28cd	34.69 ± 0.22d
Ethyl 9-decenoate	68.72 ± 7.09a	24.64 ± 0.66bc	21.33 ± 1.10c	26.44 ± 2.70b	21.78 ± 0.83bc	22.10 ± 1.02bc	20.99 ± 0.13c	21.55 ± 0.16bc	20.99 ± 1.00c
3-Methylbutyl hexanoate	15.96 ± 0.71a	9.26 ± 0.27c	9.27 ± 0.10c	10.59 ± 1.03b	8.91 ± 0.21c	9.19 ± 0.51c	8.87 ± 0.08c	9.58 ± 0.03bc	8.36 ± 1.35c
**Total other esters**	**228.71 ± 9.16a**	**104.55 ± 1.27d**	**120.16 ± 1.11c**	**163.46 ± 5.44b**	**119.87 ± 1.24c**	**100.40 ± 2.21d**	**29.86 ± 0.21g**	**78.16 ± 20.20e**	**64.05 ± 2.41f**
β-Damascenone	15.56 ± 0.95a	10.05 ± 0.66bcd	9.99 ± 1.13bcd	11.35 ± 1.05b	11.11 ± 0.37bc	9.28 ± 0.97d	9.02 ± 0.73d	9.76 ± 0.23cd	9.99 ± 0.46bcd
Benzaldehyde	4.72 ± 0.92a	1.10 ± 0.25b	0.85 ± 0.24bc	1.21 ± 0.11b	1.19 ± 0.11b	0.66 ± 0.02bc	0.58 ± 0.02bc	0.71 ± 0.10bc	0.41 ± 0.02c
2-Non-anone	1.32 ± 0.08a	0.51 ± 0.06bc	0.41 ± 0.01c	0.52 ± 0.03bc	0.50 ± 0.09bc	0.55 ± 0.01b	Nd	Nd	0.12 ± 0.01d
4-Heptanone, 2,6-dimethyl-	7.82 ± 0.99bc	10.06 ± 0.35abc	7.65 ± 0.70c	11.64 ± 1.38a	11.06 ± 2.26a	10.25 ± 1.20ab	11.29 ± 1.84a	9.24 ± 1.34abc	10.05 ± 0.94abc
**Others**	**43.28 ± 3.21a**	**33.40 ± 0.86bcd**	**27.82 ± 2.65d**	**38.39 ± 2.61ab**	**36.61 ± 4.61bc**	**32.22 ± 3.22cd**	**37.75 ± 3.18cd**	**29.66 ± 2.62d**	**31.17 ± 2.21cd**
**Total Volatile composition (mg/L)**	**684.20 ± 2.61c**	**378.85 ± 8.24f**	**498.62 ± 17.66e**	**551.95 ± 59.62d**	**389.71 ± 15.43f**	**717.55 ± 33.60c**	**715.34 ± 6.78c**	**791.42 ± 39.92b**	**841.07 ± 17.80a**

*SC, single inoculation with S. cerevisiae EC1118; ZBI-2, sequential inoculation with Z. bailii BJVI11007, followed by inoculation with EC1118 after 2 days; ZBI-3, sequential inoculation with Z. bailii BJVI11007, followed by inoculation with EC1118 after 3 days; ZBII-2, sequential inoculation with Z. bailii BJII45005, followed by inoculation with EC1118 after 2 days; ZBII-3, sequential inoculation with Z. bailii BJII45005, followed by inoculation with EC1118 after 3 days; PKI-2, sequential inoculation with P. kudriavzevii BJIV53006, followed by inoculation with EC1118 after 2 days; PKI-3, sequential inoculation with P. kudriavzevii BJIV53006, followed by inoculation with EC1118 after 3 days; PKII-2, sequential inoculation with P. kudriavzevii BJII44006, followed by inoculation with EC1118 after 2 days; PKII-3, sequential inoculation with P. kudriavzevii BJII44006, followed by inoculation with EC1118 after 3 days. Values are given as mean ± standard deviation of three replicates. Data with different letters (a, b, c, d, e, f, g, h) within each row are different according to Duncan tests (p ≤ 0.05). Nd means the compound was not detected by GC–MS in the corresponding wine sample.*

*The bold values means the total composition.*

#### C6 Alcohols and Higher Alcohols

Three C6 alcohols (1-hexanol, E-3-hexen-1-ol, Z-3-hexen-1-ol) were identified (Table 2), and their OAVs > 0.1 (Supplementary Table 4). In general, ZB could increase the total content of C6 alcohols. In C6 alcohol, the content of 1-hexanol (contributing to “herbaceous,” “grass,” and “woody” notes for wine) was the highest. PK could significantly reduce the content of 1-hexanol.

In this work, the total higher alcohols ranged from 308.54 mg/L (ZBI-2) to 631.93 mg/L (SC) (Table 2). Sequential fermentations could reduce the total higher alcohol content. 2-Methyl-1-propanol, 3-methyl-1-butanol, 3-methyl-1-pentanol, and phenylethyl alcohol were detected above their thresholds, and 1-heptanol, *leavo*-2,3-butanediol, and *meso*-2,3-butanediol were detected above their sub-thresholds (Supplementary Table 4). Sequentially fermented wines were characterized by significantly higher concentrations of 2-methyl-1-propanol (contributing to “alcohol,” “solvent,” “green,” and “bitter” notes for wine) and significantly lower amounts of 3-methyl-1-butanol, 3-methyl-1-pentanol, 4-methyl-1-pentanol, 1-heptanol, and phenylethyl alcohol. ZB could reduce the content of *leavo*-2,3-butanediol and *meso*-2,3-butanediol, while PK could significantly increase *meso*-2,3-butanediol content and decrease *leavo*-2,3-butanediol content, except PKI-2.

#### Fatty Acids

Three fatty acids (hexanoic acid, octanoic acid, and decanoic acid) were detected in this study (Table 2); among them, hexanoic acid and octanoic acid were the only two with OAVs above 0.1, and the former was up to 1 (Supplementary Table 4). The total fatty acid content was significantly lower in sequential fermentations. In PK, only a small amount of octanoic acid was detected in PKII-2 and PKII-3.

#### Esters

Esters can generally be categorized into acetate esters, fatty acid ethyl esters, and other esters. In this study, 22 esters were identified, and eight esters exceeded their thresholds (Table 2 and Supplementary Table 4), including ethyl acetate, 3-methylbutyl acetate, ethyl 2-methylpropanoate, ethyl hexanoate, ethyl heptanoate, ethyl octanoate, ethyl decanoate, and methyl decanoate.

Four acetate esters were detected in this study, and compared with SC, PK, and ZB significantly increased the content of acetate esters (Table 2). This result was reconfirmed on the third day of inoculation of *S. cerevisiae*. In esters, the content of ethyl acetate was the highest. *P. kudriavzevii* and *Z. bailli* could significantly increase the content of ethyl acetate in sequential fermentation, especially for *P. kudriavzevii* where the highest content of ethyl acetate could be increased to 369.25 mg/L. In addition, PK significantly increased the content of 3-methylbutyl acetate and 2-methylpropyl acetate. Compared with SC, sequential fermentation significantly reduced the total ethyl ester content. In all the sequentially fermented wines, the content of medium-chain fatty acid ethyl esters such as ethyl hexanoate, ethyl heptanoate, ethyl octanoate, and ethyl decanoate significantly decreased, while the content of ethyl propanoate significantly increased. *Z. Bailli* could increase the content of ethyl butanoate and ethyl 2-methylpropanoate. *P. kudriavzevii* could significantly increase the content of ethyl 2-methylpropanoate and decrease the content of ethyl butanoate. In addition to acetate and ethyl esters, five other esters were detected in this study (Table 2). The OAV of methyl decanoate was above 1 in some samples, and ethyl 9-decenoate content was higher than its subthreshold (Supplementary Table 4).

#### Others

A total of four other volatile compounds were detected in this study, including β-damascenone, benzaldehyde, 2-nonanone, and 4-heptanone, 2,6-dimethyl- (Table 2). β-Damascenone contributed the “sweet,” “exotic flower,” and “stewed apple” aromatic notes to the wine. Compared with SC, sequential fermentation reduced the content of β-damascenone, but the OAV value in pure fermentation was still much higher than 1 (Supplementary Table 4). In addition, 2-nonanone content was higher than its subthreshold, which contributed to the fruity aroma of the wine.

### Principal Component Analysis and Hierarchical Cluster Analysis of Wine Aroma Compounds in Different Fermentations

**FIGURE 3:**
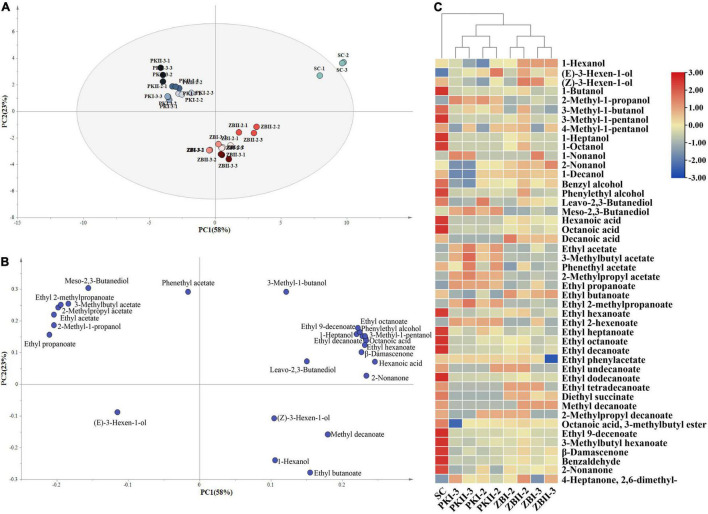
Principal component analysis (PCA) and hierarchical cluster analysis (HCA) of wine volatile components in different fermentations. **(A,B)** Principal component analysis (PCA) biplots of wines resulting from 26 volatile compounds (OAV above 0.1) by different fermentation methods after alcoholic fermentation. **(C)** Hierarchical clustering and heatmap visualization of volatile compounds of wine samples in different fermentations.

To highlight the differences in the fermentation of different inoculation strategies and to identify the effects of these treatments on volatile compounds, PCA was performed on 27 volatile compounds with OAV above 0.1. As shown in Figures 3A,B, the first two components accounted for 81% (58% for PC1 and 23% for PC2) of the total variance. The PCs were roughly distinguished by wine samples fermented by different inoculation strategies. Wines produced by SC were separated from the other wines by PC1. The main components responsible for this separation were 3-methyl-1-butanol, *leavo*-2,3-butanediol, 1-heptanol, phenylethyl alcohol, 3-methyl-1-pentanol, octanoic acid, hexanoic acid, ethyl heptanoate, ethyl octanoate, ethyl hexanoate, ethyl decanoate, and β-damascenone. PC2 separated PK from ZB, mainly by 2-methyl-1-propanol, *meso*-2,3-butanediol, ethyl acetate, 2-methylpropyl acetate, 3-methylbutyl acetate, ethyl propanoate, and ethyl 2-methylpropanoate. These data indicate that the volatile compounds of *S. cerevisiae* can be further affected by sequential fermentation with non-*Saccharomyces* yeast.

The dendrogram of Hierarchical Cluster Analysis (HCA) was used to visualize the differences in volatile compounds among different inoculation strategies (Figure 3C). The results showed that the samples can be divided into two categories: pure fermentation and sequential fermentations. Notably, the sequential fermentation of two non-*Saccharomyces* yeast strains of the same species was divided into two groups according to the inoculation time of *S. cerevisiae*, which indicates the inoculation time of *S. cerevisiae* had a significant effect on the volatile compounds of sequentially fermented wine.

### Sensory Evaluation

**FIGURE 4:**
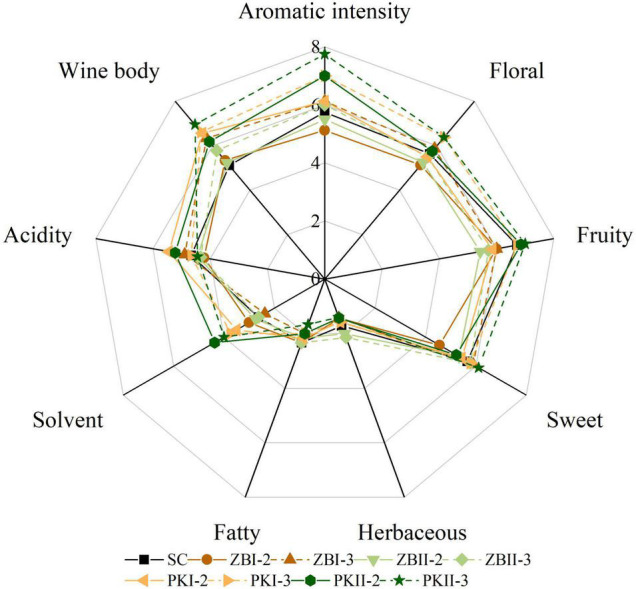
Sensory evaluation of final *Cabernet Sauvignon* wines. SC, black solid line; ZBI-2, brown solid line; ZBI-3, brown dotted line; ZBII-2, olive green solid line; ZBII-3, olive green dotted line; PKI-2, orange solid line; PKI-3, orange dotted line; PKII-2, deep green solid line; PKII-3, deep green dotted line.

The sensory evaluation results of the wine samples were derived from 12 assessors, and the aroma radar map was drawn as shown in Figure 4. It can be noticed that yeast strains and inoculation strategies have a great influence on the aroma characteristics of wine. The score of “aromatic intensity” and “wine body” in sequential fermented wines was higher than those of SC (except ZBI-2 and ZBII-2). Compared with SC, PKI-3 and PKII-3 had a high aroma note for floral, fruity and sweet, and a low aroma note for herbaceous and fatty. In addition, PK had a high aroma note for solvent, in agreement with their high levels of the ethyl acetate; ZB had a low aroma note for fruity, in agreement with their low levels of higher alcohols and esters. It was noteworthy that, PKII-3 had a high aroma note for floral, fruity, sweet, and aromatic intensity, and it also had a high score of “wine body.” These results showed that non-*Saccharomyces* yeasts selected from the Baijiu fermentation environment have the potential to improve the flavor and quality of wine.

## Discussion

To explore the application potential for Baijiu non-*Saccharomyces* yeast strains in wine fermentation, the effect of four Baijiu non-*Saccharomyces* yeast strains (two *Z. bailii* and two *P. kudriavzevii*) inoculated with one *S. cerevisiae* (commercial strain EC1118) on the physicochemical parameters and volatile compositions of wine was investigated in this study.

The contribution of yeast to wine volatile compounds is largely dependent on the persistence of strains and the number of yeast cells during alcoholic fermentation. In this study, it was found that there was no obvious antagonism between *S. cerevisiae* and *Z. bailli* or *P. kudriavzevii*, and all strains could be detected at the end of alcohol fermentation. The fermentation process of sequential fermentations was significantly longer than that of *S. cerevisiae* pure fermentation, because *Z. bailli* and *P. kudriavzevii* consumed glucose and fructose significantly slower than *S. cerevisiae* (Supplementary Figure 1). The acidity of grape juice and wine directly affects their sensory quality and physical, biochemical, and microbial stability (Swiegers and Pretorius, 2005). Acetic acid usually accounts for about 90% of the volatile acids in wine (Swiegers and Pretorius, 2005). At the concentration of 0.7–1.1 g/L, acetic acid imparts an unpleasant smell to wine, and the best concentration is 0.2–0.7 g/L (Swiegers and Pretorius, 2005). In this study, sequential fermentation (except for ZBI-2 and ZBI-3) could significantly reduce the acetic acid content. Previous studies have found that *Z. bailii* can produce acetic acid (Xu et al., 2017b). In this study, the two *Z. bailii* strains showed different characteristics of acetic acid production, which may be related to the differences between strains. Non-volatile organic acids have a direct impact on the quality of wine; the imbalance of this component will affect its physicochemical and sensory properties (Gawel et al., 2007) and change its microbial properties (Delcourt et al., 1995; Pretorius, 2000). In this study, the malic acid content of sequential fermentation was higher and the lactic acid content was lower, which may lower the quality of wine but can be improved by malolactic fermentation. Our results clearly demonstrate that sequential fermentation of *Z. bailli* and *S. cerevisiae* could significantly increase the content of succinic acid in agreement with the research results of Zhu et al. (2020). It may be due to the non-*Saccharomyces* yeast can exhibit low activity through acetaldehyde pathway which trigger an important redistribution of fluxes through the central metabolic network (Englezos et al., 2018). The high level of acetyl-CoA in *Z. bailli*, leads to an increase in the level of α-ketoglutarate and a significant increase in succinic acid content.

Yeast is one of the important factors affecting wine fermentation aroma. Our results demonstrate that two non-*Saccharomyces* yeast strains of the same species had common effects on wine volatile compounds. Consistent with previous literature (Shi et al., 2019), we found that the inoculation time of *S. cerevisiae* affected the volatile compositions of the wines. Notably, through HCA (Figure 3C), we found in sequential fermentations, compared with the difference between the two non-*Saccharomyces* yeast strains of the same species, the inoculation time of *S. cerevisiae* had a greater effect on wine volatile compounds. Higher alcohols are produced by the decarboxylation and dehydrogenation of α-ketoacids through the Ehrlich pathway and Harris pathway (Wang et al., 2020). When the concentration is low (<300 mg/L), they can increase the complexity of wine aroma (Swiegers and Pretorius, 2005), while at higher concentrations (> 400 mg/L), higher alcohols weaken the fresh fruit aroma, enhance the pepper characteristics of young red wine, and harm the overall flavor of wine (Aznar et al., 2003; San-Juan et al., 2011). In this study, the content of total higher alcohols in SC was as high as 631.93 mg/L (Table 2), which mainly consisted of 2-methyl-1-propanol (47.59 mg/L), isoamyl alcohol (270.47 mg/L), and phenylethyl alcohol (248.34 mg/L). Sequential fermentation of non-*Saccharomyces* yeasts and *S. cerevisiae* significantly reduced the content of higher alcohols (308.54–499.66 mg/L). The total amounts of higher alcohols in all treatments were above 300 mg/L (Table 2), which may be related to the grapes used in this experiment. 2-Methyl-1-propanol and 3-methyl-1-butanol are produced by yeasts during alcoholic fermentation through the conversion of valine and isoleucine, respectively, *via* the Ehrlich pathway (Hazelwood et al., 2008). It was worth noting that compared with SC, the content of 2-methyl-1-propanol in all sequentially fermented wines significantly increased, while the content of isoamyl alcohol significantly decreased. It may be due to changes in acetyl-coenzyme (acetyl-CoA) availability, which is required for the conversion of α-ketoisovalerate, the precursor of 2-methyl-1-propanol, into α-ketoisocaproic, the precursor of isoamyl alcohol (Hazelwood et al., 2008). The concentration of phenylethyl alcohol is higher than its threshold, which contributes to the rose aroma of wine (Tristezza et al., 2016). In this study, the OAV of phenylethyl alcohol in all samples was above 1. Sequential fermentation significantly reduced the content of phenylethyl alcohol and may reduce the negative effect of high higher alcohol content on wine.

Volatile fatty acids are essential to the aroma of the wine. When the content of volatile fatty acids is at the subthreshold, it will have a positive effect on the aroma of wine, and when it exceeds the threshold, it will spoil the wine aroma (Swiegers and Pretorius, 2005). In this study, the OAV of hexanoic acid > 1 in SC may bring a fatty flavor to the wine and adversely affect the aroma of the wine. Sequential fermentation reduced the content of hexanoic acid, and the OAV of hexanoic acid in ZB was more than 0.1, which could render a “cheese” note to the wine. In addition, the volatile fatty acid is the precursor of fatty acid ethyl ester synthesis (Hernández-Orte et al., 2006). Sequential fermentation reduces the content of volatile fatty acids, leading to the decrease of fatty acid ethyl ester content.

Yeast strains showed high specificity in total ester yield and acetate and ethyl ester patterns, which led to sensory differences in wine (Soles et al., 1982). In this study, *Z. bailli* and *P. kudriavzevii* were associated with a higher production of acetate esters and lower production of ethyl esters. Ethyl acetate is the main ester in wine (Moreira et al., 2011), and it can be biosynthesized by acetyl-CoA and ethanol through the reaction catalyzed by alcohol acyltransferase (Shi et al., 2021), which adds a pleasant fruit aroma to wine at low concentrations. However, when the concentration of ethyl acetate is higher than 150 mg/L, the chemical odor of varnish may damage the aroma of wine (Peinado et al., 2004). In this study, the ethyl acetate concentration of ZB was between 48.87 mg/L and 155.85 mg/L, and the ethyl acetate concentration of PK was between 248.01 mg/L and 369.25 mg/L, which was much higher than 150 mg/L and would thus have a negative effect on wine aroma (Comitini et al., 2011; Mateo and Maicas, 2016). In the future, when making wine with *P. kudriavzevii*, we can try to reduce the negative effects of very high concentrations of ethyl acetate by reducing the inoculation amount of *P. kudriavzevii* or adopting the strategy of simultaneous inoculation. In addition, compared with SC, PK could also significantly increase the content of 3-methylbutyl acetate and 2-methylpropyl acetate in agreement with the observations of Padilla et al. (2016) and Luan et al. (2018). Ethyl ester is another important group of esters in wine, and they are produced during yeast fermentation through ethanolysis of acyl-CoA that is formed during fatty acid synthesis or degradation (Swiegers and Pretorius, 2005). In this study, compared with SC, the ethyl ester content of wine fermented in the sequence was lower, mainly because the content of medium-chain fatty acid esters (ethyl esters of fatty acids with 6–12 carbon atoms) was reduced by sequential fermentation. It may be caused by the decrease of fatty acid content as its precursor (Saerens et al., 2010). In addition, compared with SC, sequential fermentation could significantly increase the content of ethyl propionate and ethyl 2-methylpropanoate, and ZB could also significantly increase the content of ethyl butanoate, rendering “banana” and “pear” notes to the wine.

## Conclusion

In summary, these experiments indicate that there was no obvious antagonism between *S. cerevisiae* and *Z. bailli* or *P. kudriavzevii* in sequential fermentations, and all strains could be detected at the end of alcoholic fermentation. Compared with the pure fermentation of *S. cerevisiae*, the sequential fermentation of *Z. bailii* and *S. cerevisiae* could significantly reduce the content of higher alcohols and ethyl esters and increase the content of acetate esters; the sequential fermentation of *P. kudriavzevii* and *S. cerevisiae* could significantly reduce the content of C6 alcohols, total higher alcohols, and ethyl esters and significantly increased the contents of acetate esters (especially ethyl acetate and 3-methylbutyl acetate). Sequential fermentation of Baijiu non-*Saccharomyces* yeast and *S. cerevisiae* improved the flavor and quality of wine due to the higher ester content and lower concentration of higher alcohols and fatty acids, non-*Saccharomyces* yeasts selected from the Baijiu fermentation environment have potential applications in winemaking, which could provide a new strategy to improve wine flavor and quality.

## Data Availability Statement

The original contributions presented in the study are included in the article/Supplementary Material, further inquiries can be directed to the corresponding author/s.

## Author Contributions

X-LW and Y-FC designed the experiments. R-RL, MX, JZ, Y-JL, and C-HS conducted the experiments. R-RL, MX, JZ, Y-JL, C-HS, and HW analyzed the experimental data. R-RL wrote the manuscript. X-WG and D-GX contributed to data curation. All authors contributed to the article and approved the submitted version.

## Conflict of Interest

The authors declare that the research was conducted in the absence of any commercial or financial relationships that could be construed as a potential conflict of interest.

## Publisher’s Note

All claims expressed in this article are solely those of the authors and do not necessarily represent those of their affiliated organizations, or those of the publisher, the editors and the reviewers. Any product that may be evaluated in this article, or claim that may be made by its manufacturer, is not guaranteed or endorsed by the publisher.

## References

[B1] Aznar M. López R. Cacho J. Ferreira V. (2003). Prediction of aged red wine aroma properties from aroma chemical composition. Partial least squares regression models. *J. Agric. Food Chem*. 51 2700–2707. 10.1021/jf026115z 12696960

[B2] Benito Á Calderón F. Benito S. (2019). The Influence of Non-*Saccharomyces* species on wine fermentation quality parameters. *Fermentation* 5:54. 10.3390/fermentation5030054

[B3] Benito S. (2018). The impact of *Torulaspora delbrueckii* yeast in winemaking. *Appl. Microbiol. Biotechnol*. 102 3081–3094. 10.1007/s00253-018-8849-0 29492641

[B4] Cai J. Zhu B. Wang Y. Lu L. Lan Y. Reeves M. (2014). Influence of pre-fermentation cold maceration treatment on aroma compounds of *Cabernet Sauvignon* wines fermented in different industrial scale fermenters. *Food Chem*. 154 217–229. 10.1016/j.foodchem.2014.01.003 24518336

[B5] Chen C. Chen H. Zhang Y. Thomas H. Xia R. (2020). Tbtools: an integrative toolkit developed for interactive analyses of big biological data. *Mol. Plant* 13 1194–1202. 10.1016/j.molp.2020.06.009 32585190

[B6] Ciani M. Comitini F. Mannazzu I. Domizio P. (2010). Controlled mixed culture fermentation: a new perspective on the use of non-*Saccharomyces* yeasts in winemaking. *FEMS Yeast Res.* 10 123–133. 10.1111/j.1567-1364.2009.00579.x 19807789

[B7] Comitini F. Gobbi M. Domizio P. Romani C. Lencioni L. Mannazzu I. (2011). Selected non-*Saccharomyces* wine yeasts in controlled multistarter fermentations with *Saccharomyces cerevisiae*. *Food Microbiol*. 28 873–882. 10.1016/j.fm.2010.12.001 21569929

[B8] Delcourt F. Taillandier P. Vidal F. Strehaiano P. (1995). Influence of pH, malic acid and glucose concentrations on malic acid consumption by *Saccharomyces cerevisiae*. *Appl. Microbiol. Biotechnol*. 43 321–324. 10.1007/BF0017283222 7612251

[B9] Englezos V. Cocolin L. Rantsiou K. Ortiz-Julien A. Bloem A. Dequin S. (2018). Specific phenotypic traits of *Starmerella bacillaris* related to nitrogen source consumption and central carbon metabolite production during wine fermentation. *Appl. Environ. Microbiol.* 84:e00797–18. 10.1128/aem.00797-18 29858207 PMC6070767

[B10] Escudero A. Campo E. Farina L. Cacho J. Ferreira V. (2007). Analytical characterization of the aroma of five premium red wines. Insights into the role of odor families and the concept of fruitiness of wines. *J. Agric. Food Chem.* 55 4501–4510. 10.1021/jf0636418 17488088

[B11] Fan W. Qian M. C. (2006). Characterization of aroma compounds of Chinese “Wuliangye” and “Jiannanchun” liquors by aroma extract dilution analysis. *J. Agr. Food Chem*. 54 2695–2704. 10.1021/jf052635t 16569063

[B12] Garavaglia J. de Souza Schneider R. D. C. Mendes S. D. C. Welke J. E. Zini C. A. Caramão E. B. (2015). Evaluation of *Zygosaccharomyces bailii* BCV 08 as a co-starter in wine fermentation for the improvement of ethyl esters production. *Microbiol. Res*. 173 59–65. 10.1016/j.micres.2015.02.002 25801972

[B13] Gawel R. Francis L. Waters E. J. (2007). Statistical correlations between the in-mouth textural characteristics and the chemical composition of Shiraz wines. *J. Agric. Food Chem*. 55 2683–2687. 10.1021/jf0633950 17348678

[B14] Guth H. (1997). Quantification and sensory studies of character impact odorants of different white wine varieties. *J. Agric. Food Chem*. 45 3027–3032. 10.1021/jf970280a 10552816

[B15] Hazelwood L. A. Daran J. M. Van Maris A. J. Pronk J. T. Dickinson J. R. (2008). The Ehrlich pathway for fusel alcohol production: a century of research on *Saccharomyces cerevisiae* metabolism. *Appl. Environ. Microb*. 74 2259–2266. 10.1128/AEM.02625-07 18281432 PMC2293160

[B16] Hernández-Orte P. Ibarz M. J. Cacho J. Ferreira V. (2006). Addition of amino acids to grape juice of the Merlot variety: effect on amino acid uptake and aroma generation during alcoholic fermentation. *Food Chem*. 98 300–310. 10.1016/j.foodchem.2005.05.073

[B17] Jiang J. Liu Y. Li H. Yang Q. Wu Q. Chen S. (2019). Modeling and regulation of higher alcohol production through the combined effects of the C/N ratio and microbial interaction. *J. Agric. Food Chem*. 67 10694–10701. 10.1021/acs.jafc.9b04545 31476866

[B18] Kurita O. (2008). Increase of acetate ester-hydrolysing esterase activity in mixed cultures of *Saccharomyces cerevisiae* and *Pichia anomala*. *J. Appl. Microbiol*. 104 1051–1058. 10.1111/j.1365-2672.2007.03625.x 17976172

[B19] Leixà J. Martín V. Portillo M. D. C. Carrau F. Beltran G. Mas A. (2016). Comparison of fermentation and wines produced by inoculation of *Hanseniaspora vineae* and *Saccharomyces cerevisiae*. *Front. Microbiol*. 7:338. 10.3389/fmicb.2016.00338 27014252 PMC4792884

[B20] Li N. Wang Q. Q. Xu Y. H. Li A. H. Tao Y. S. (2020). Increased glycosidase activities improved the production of wine varietal odorants in mixed fermentation of *P. fermentans* and high antagonistic *S. cerevisiae*. *Food Chem*. 332:127426. 10.1016/j.foodchem.2020.127426 32619948

[B21] Li R. R. Sun Y. X. (2019). Effects of honey variety and non-*Saccharomyces cerevisiae* on the flavor volatiles of mead. *J. Am. Soc. Brew. Chem*. 77 40–53. 10.1080/03610470.2018.1546072

[B22] Liu P. Xiong X. Wang S. Miao L. (2017). Population dynamics and metabolite analysis of yeasts involved in a Chinese miscellaneous flavor liquor fermentation. *Ann. Microbiol*. 67 553–565. 10.1007/s13213-017-1286-y

[B23] Luan Y. Zhang B. Q. Duan C. Q. Yan G. L. (2018). Effects of different pre-fermentation cold maceration time on aroma compounds of *Saccharomyces cerevisiae* co-fermentation with *Hanseniaspora opuntiae* or *Pichia kudriavzevii*. *LWT Food Sci. Technol*. 92 177–186. 10.1016/j.lwt.2018.02.004

[B24] Ma D. Yan X. Wang Q. Zhang Y. Tao Y. S. (2017). Performance of selected *P. fermentans* and its excellular enzyme in co-inoculation with *S. cerevisiae* for wine aroma enhancement. *LWT Food Sci. Technol*. 86 361–370. 10.1016/j.lwt.2017.08.018

[B25] Mateo J. J. Maicas S. (2016). Application of non-*Saccharomyces* yeasts to wine-making process. *Fermentation* 2:14. 10.3390/fermentation2030014

[B26] Moreira N. Pina C. Mendes F. Couto J. A. Hogg T. Vasconcelos I. (2011). Volatile compounds contribution of *Hanseniaspora guilliermondii* and *Hanseniaspora uvarum* during red wine vinifications. *Food Control* 22 662–667. 10.1016/j.foodcont.2010.07.025

[B27] Padilla B. Gil J. V. Manzanares P. (2016). Past and future of non-*Saccharomyces* yeasts: from spoilage microorganisms to biotechnological tools for improving wine aroma complexity. *Front. Microbiol*. 7:411. 10.3389/fmicb.2016.00411 27065975 PMC4814449

[B28] Palma M. de Canaveira Roque F. Guerreiro J. F. Mira N. P. Queiroz L. Sá-Correia I. (2015). Search for genes responsible for the remarkably high acetic acid tolerance of a *Zygosaccharomyces bailii*-derived interspecies hybrid strain. *BMC Genomics* 16:1070. 10.1186/s12864-015-2278-6 26673744 PMC4681151

[B29] Peinado R. A. Mauricio J. C. Medina M. Moreno J. J. (2004). Effect of *Schizosaccharomyces pombe* on aromatic compounds in dry sherry wines containing high levels of gluconic acid. *J. Agric. Food Chem*. 52 4529–4534. 10.1021/jf049853r 15237962

[B30] Pretorius I. S. (2000). Tailoring wine yeast for the new millennium: novel approaches to the ancient art of winemaking. *Yeast* 16 675–729. 10.1002/1097-0061(20000615)16:8<675::AID-YEA585>3.0.CO;2-B 10861899

[B31] Renault P. Coulon J. de Revel G. Barbe J. C. Bely M. (2015). Increase of fruity aroma during mixed *Torulaspora delbrueckii*/*Saccharomyces cerevisiae* wine fermentation is linked to specific esters enhancement. *Int. J. Food Microbiol*. 207 40–48. 10.1016/j.ijfoodmicro.2015.04.037 26001522

[B32] Romano P. Suzzi G. (1993). Higher alcohol and acetoin production by *Zygosaccharomyces* wine yeasts. *J. Appl. Bacteriol*. 75 541–545. 10.1111/j.1365-2672.1993.tb01592.x

[B33] Ryan D. Prenzler P. Saliba A. Scollary G. (2008). The significance of low impact odorants in global odour perception. *Trends Food Sci. Technol*. 19 383–389. 10.1016/j.tifs.2008.01.007

[B34] Saerens S. M. Delvaux F. R. Verstrepen K. J. Thevelein J. M. (2010). Production and biological function of volatile esters in *Saccharomyces cerevisiae*. *Microbiol. Biotechnol*. 3 165–177. 10.1111/j.1751-7915.2009.00106.x 21255318 PMC3836583

[B35] San-Juan F. Ferreira V. Cacho J. Escudero A. (2011). Quality and aromatic sensory descriptors (mainly fresh and dry fruit character) of spanish red wines can be predicted from their aroma-active chemical composition. *J. Agric. Food Chem*. 59 7916–7924. 10.1021/jf1048657 21627324

[B36] Shi W. K. Li J. Chen Y. Liu X. H. Chen Y. F. Guo X. W. (2021). Metabolic engineering of *Saccharomyces cerevisiae* for ethyl acetate biosynthesis. *ACS Synth. Biol*. 10 495–504. 10.1021/acssynbio.0c00446 33576609

[B37] Shi W. K. Wang J. Chen F. S. Zhang X. Y. (2019). Effect of *Issatchenkia terricola* and *Pichia kudriavzevii* on wine flavor and quality through simultaneous and sequential co-fermentation with *Saccharomyces cerevisiae*. *LWT Food Sci. Technol*. 116:108477. 10.1016/j.lwt.2019.108477

[B38] Soles R. M. Ough C. S. Kunkee R. E. (1982). Ester concentration differences in wine fermented by various species and strains of yeasts. *Am. J. Enol. Viticult*. 33 94–98. 10.1016/0141-4607(82)90006-3

[B39] Stratford M. Steels H. Nebe-von-Caron G. Novodvorska M. Hayer K. Archer D. B. (2013). Extreme resistance to weak-acid preservatives in the spoilage yeast *Zygosaccharomyces bailii*. *Int. J Food Microbiol*. 166 126–134. 10.1016/j.ijfoodmicro.2013.06.025 23856006 PMC3759830

[B40] Swiegers J. H. Pretorius I. S. (2005). Yeast modulation of wine flavor. *Adv. Appl. Microbiol*. 57 131–175. 10.1016/S0065-2164(05)57005-9 16002012

[B41] Tristezza M. Tufariello M. Capozzi V. Spano G. Mita G. Grieco F. (2016). The oenological potential of *Hanseniaspora uvarum* in simultaneous and sequential co-fermentation with *Saccharomyces cerevisiae* for industrial wine production. *Front. Microbiol*. 7:670. 10.3389/fmicb.2016.00670 27242698 PMC4860541

[B42] Wang S. Wu Q. Nie Y. Wu J. Xu Y. (2019). Construction of synthetic microbiota for reproducible flavor compound metabolism in Chinese light-aroma-type liquor produced by solid-state fermentation. *Appl. Environ. Microbiol.* 85:e03090–18. 10.1128/AEM.03090-18 30850432 PMC6498162

[B43] Wang Y. P. Wei X. Q. Guo X. W. Xiao D. G. (2020). Effect of the deletion of genes related to amino acid metabolism on the production of higher alcohols by *Saccharomyces cerevisiae*. *BioMed. Res. Int.* 2020:6802512. 10.1155/2020/6802512 33204707 PMC7665916

[B44] Xu Y. Sun B. Fan G. Chao T. Li X. (2017a). The brewing process and microbial diversity of strong flavour Chinese spirits: a review. *J. Inst. Brew*. 123 5–12. 10.1002/jib.404

[B45] Xu Y. Zhi Y. Wu Q. Du R. (2017b). *Zygosaccharomyces bailii* is a potential producer of various flavor compounds in Chinese Maotai-flavor liquor fermentation. *Front. Microbiol*. 8:2609. 10.3389/fmicb.2017.02609 29312273 PMC5744019

[B46] Xu Y. Q. Zhao J. G. Liu X. Zhang C. S. Zhao Z. G. Li X. T. (2022). Flavor mystery of Chinese traditional fermented baijiu: the great contribution of ester compounds. *Food Chem*. 369:130920. 10.1016/j.foodchem.2021.130920 34461518

[B47] Zhang B. Q. Luan Y. Duan C. Q. Yan G. L. (2018). Use of *Torulaspora delbrueckii* co-fermentation with two *Saccharomyces cerevisiae* strains with different aromatic characteristic to improve the diversity of red wine aroma profile. *Front. Microbiol*. 9:606. 10.3389/fmicb.2018.00606 29674999 PMC5895779

[B48] Zhang B. Q. Tang C. Yang D. Q. Liu H. Xue J. Duan C. H. (2022). Effects of three indigenous non-*Saccharomyces* yeasts and their pairwise combinations in co-fermentation with *Saccharomyces cerevisiae* on volatile compounds of Petit Manseng wines. *Food Chem.* 368:130807. 10.1016/j.foodchem.2021.130807 34411859

[B49] Zhu X. Navarro Y. Mas A. Torija M. J. Beltran G. (2020). A rapid method for selecting non-*Saccharomyces* strains with a low ethanol yield. *Microorganisms* 8:658. 10.3390/microorganisms8050658 32369912 PMC7284643

