# Supplementary material for: Application Potential of Baijiu Non-Saccharomyces Yeast in Winemaking Through Sequential Fermentation With Saccharomyces cerevisiae
Source: Front Microbiol. 2022 May 30;13:902597. doi: 10.3389/fmicb.2022.902597 (PMC9196592; doi:10.3389/fmicb.2022.902597)
Supplement: Supplementary file 1 [file Data_Sheet_1.docx]

Supplementary Material

# Supplementary Tables

**Supplementary Table S1.** The 26S rDNA gene D1/D2 sequence of the four indigenous non-*Saccharomyces* yeast strains was used in this study.

| Strain | 26S rDNA gene D1/D2 sequence |
| --- | --- |
| BJVI11007 | CGGAGGAAAAGAAACCAACCGGGATTGCCTTAGTACGGCGAGTGAAGCGGCAAAAGCTCAAATTTGAAATCTGGTACCTTCGGTGCCCGAGTTGTAATTTGTAGAAGGCGACTCTGGGGCTGGTCCTTGTCTATGTTCCTTGGAACAGGACGTCATGGAGGGTGAGAATCCCGTATGGCGAGGATCCCAGTTCTTTGTAGAGTGCCTTCGAAGAGTCGAGTTGTTTGGGAATGCAGCTCTAAGTGGGTGGTAAATTCCATCTAAAGCTAAATATTGGCGAGAGACCGATAGCGAACAAGTACAGTGATGGAAAGATGAAAAGAACTTTGAAAAGAGAGTGAAAAAGTACGTGAAATTGTTGAAAGGGAAGGGCATTTGATCAGACATGGTGTTTTGCGCCCCTCGCCTCTCGTGGGTGGGGGAATCTCGCAGCTCACTGGGCCAGCATCAGTTTTGGCGGCAGGATAAATCCCTGGGAATGTAGCTCTACCACTTCGTGGCGGACGAACTTATAGTCCAGGGGAATACTGCCAGCTGGGACTGAGGAATGCGACTTTTAGTCAAGGATGCTGGCATAATGGTTATATGCCGCCCGTCTTGAAACACG |
| BJII45005 | AGCGGAGGAAAAGAAACCAACCGGGATTGCCTTAGTACGGCGAGTGAAGCGGCAAAAGCTCAAATTTGAAATCTGGTACCTTCGGTGCCCGAGTTGTAATTTGTAGAAGGCGACTCTGGGGCTGGTCCTTGTCTATGTTCCTTGGAACAGGACGTCATGGAGGGTGAGAATCCCGTATGGCGAGGATCCCAGTTCTTTGTAGAGTGCCTTCGAAGAGTCGAGTTGTTTGGGAATGCAGCTCTAAGTGGGTGGTAAATTCCATCTAAAGCTAAATATTGGCGAGAGACCGATAGCGAACAAGTACAGTGATGGAAAGATGAAAAGAACTTTGAAAAGAGAGTGAAAAAGTACGTGAAATTGTTGAAAGGGAAGGGCATTTGATCAGACATGGTGTTTTGCGCCCCTCGCCTCTCGTGGGTGGGGGAATCTCGCAGCTCACTGGGCCAGCATCAGTTTTGGCGGCAGGATAAATCCCTGGGAATGTAGCTCTACCACTTCGTGGCGGACGAACTTATAGTCCAGGGGAATACTGCCAGCTGGGACTGAGGAATGCGACTTTTAGTCAAGGATGCTGGCATAATGGTTATATGCCGCCCGTCTTGAAACACGGGACCA |
| BJIV53006 | GCGGAGGAAAAGAAACCAACAGGGATTGCCTCAGTAGCGGCGAGTGAAGCGGCAAGAGCTCAGATTTGAAATCGTGCTTTGCGGCACGAGTTGTAGATTGCAGGTTGGAGTCTGTGTGGAAGGCGGTGTCCAAGTCCCTTGGAACAGGGCGCCCAGGAGGGTGAGAGCCCCGTGGGATGCCGGCGGAAGCAGTGAGGCCCTTCTGACGAGTCGAGTTGTTTGGGAATGCAGCTCCAAGCGGGTGGTAAATTCCATCTAAGGCTAAATACTGGCGAGAGACCGATAGCGAACAAGTACTGTGAAGGAAAGATGAAAAGCACTTTGAAAAGAGAGTGAAACAGCACGTGAAATTGTTGAAAGGGAAGGGTATTGCGCCCGACATGGGGATTGCGCACCGCTGCCTCTCGTGGGCGGCGCTCTGGGCTTTCCCTGGGCCAGCATCGGTTCTTGCTGCAGGAGAAGGGGTTCTGGAACGTGGCTCTTCGGAGTGTTATAGCCAGGGCCAGATGCTGCGTGCGGGGACCGAGGACTGCGGCCGTGTAGGTCACGGATGCTGGCAGAACGGCGCAACACCGCCCGTCTTGAAACACGGACCA |
| BJII44006 | AAGCGGAGGAAAAGAAACCAACAGGGATTGCCTCAGTAGCGGCGAGTGAAGCGGCAAGAGCTCAGATTTGAAATCGTGCTTTGCGGCACGAGTTGTAGATTGCAGGTTGGAGTCTGTGTGGAAGGCGGTGTCCAAGTCCCTTGGAACAGGGCGCCCAGGAGGGTGAGAGCCCCGTGGGATGCCGGCGGAAGCAGTGAGGCCCTTCTGACGAGTCGAGTTGTTTGGGAATGCAGCTCCAAGCGGGTGGTAAATTCCATCTAAGGCTAAATACTGGCGAGAGACCGATAGCGAACAAGTACTGTGAAGGAAAGATGAAAAGCACTTTGAAAAGAGAGTGAAACAGCACGTGAAATTGTTGAAAGGGAAGGGTATTGCGCCCGACATGGGGATTGCGCACCGCTGCCTCTCGTGGGCGGCGCTCTGGGCTTTCCCTGGGCCAGCATCGGTTCTTGCTGCAGGAGAAGGGGTTCTGGAACGTGGCTCTTCGGAGTGTTATAGCCAGGGCCAGATGCTGCGTGCGGGGACCGAGGACTGCGGCCGTGTAGGTCACGGATGCTGGCAGAACGGCGCAACACCGCCCGTCTTGAAACACGGACCA |

**Supplementary Table S2.** The physicochemical parameters detected in our work with their Quantitative standards, Manufacturers of quantitative standards, Calibration curves, R^2^ value, and linear range.

| Compounds | Quantitative standard | Manufacturers | Calibration curves | R^2^ | Linear rang (g/L) |
| --- | --- | --- | --- | --- | --- |
| Ethanol | Ethanol | Aladdin | y=117003x+1173.5 | 0.9999 | 0.2508~25.08 |
| Glycerol | Glycerol | Aladdin | y=270870x-4964.1 | 0.9999 | 0.0411~4.11 |
| Glucose | D-(+)-Glucose | Aladdin | y=332555x-16377 | 0.9999 | 0.1004~10.04 |
| Fructose | Fructose | Aladdin | y=393298x-10334 | 0.9999 | 0.1002~~10.02 |
| Citric acid | Citric acid | Solarbio | y=2.8827x-47.012 | 0.9998 | 0.1002~2.004 |
| Tartaric acid | L-Malic acid | Aladdin | y=4.4545x-64.582 | 0.9998 | 0.10005~2.001 |
| Malic acid | L-Malic acid | Solarbio | y=2.2559x-37.83 | 0.9998 | 0.1012~2.024 |
| Succinic acid | Succinic acid | Sigma-Aldrich | y=2.4104x-90.111 | 0.9983 | 0.1014~2.028 |
| Lactic acid | L-Lactic acid | Solarbio | y=130776x-1261.2 | 0.9999 | 0.02~2.00 |
| Acetic acid | Acetic acid | Aladdin | y = 2.5662x-70.977 | 0.9972 | 0.05~1.00 |

**Supplementary Table S3.** The odor-active compounds detected in our work with their Quantitative standards, CAS of quantitative standards, Calibration curves, R^2^ value, and linear range.

| Compounds | Quantitative standard | CAS | Calibration curves | R^2^ | Linear rang (mg/L) |
| --- | --- | --- | --- | --- | --- |
| 1-Hexanol | 1-Hexanol | 111273 | y=0.5802x-0.0811 | 0.9994 | 0.02~20.02 |
| (E)-3-Hexen-1-ol | (Z)-3-Hexen-1-ol | 928961 | y=0.259x-0.0434 | 0.9992 | 0.01~20.004 |
| (Z)-3-Hexen-1-ol | (Z)-3-Hexen-1-ol | 928961 | y=0.259x-0.0434 | 0.9992 | 0.01~20.004 |
| 1-Butanol | 1-Butanol | 71363 | y=0.0105x+0.0249 | 0.9993 | 0.1~50.22 |
| 2-Methyl-1-propanol | 2-Methyl-1-propanol | 78831 | y= 0.0122x-0.011 | 0.9997 | 0.2~200.18 |
| 3-Methyl-1-butanol | 3-Methyl-1-butanol | 123513 | y=0.0632x+0.0485 | 0.9998 | 0.2~400.464 |
| 3-Methyl-1-pentanol | 3-Methyl-1-pentanol | 589355 | y=0.3445x-0.0638 | 0.9909 | 0.02~20.015 |
| 4-Methyl-1-pentanol | 3-Methyl-1-pentanol | 589355 | y=0.3445x-0.0638 | 0.9909 | 0.02~20.015 |
| 1-Heptanol | 1-Heptanol | 111706 | y=0.9867x+0.727 | 0.9808 | 0.01~20.02 |
| 1-Octanol | 1-Octanol | 111875 | y =7.0755x+0.0517 | 0.9978 | 0.0004~1 |
| 1-Nonanol | 1-Nonanol | 143088 | y=18.094x-0.2784 | 0.9998 | 0.005~1 |
| 2-Nonanol | 2-Nonanol | 628999 | y=17.16x-0.0498 | 0.9989 | 0.0002~0.399 |
| Benzyl alcohol | Benzyl alcohol | 100516 | y=0.0859x+0.0172 | 0.9976 | 0.02~24.96 |
| Phenylethyl alcohol | Phenylethyl alcohol | 60128 | y=0.134x+0.356 | 0.9981 | 0.3~299.88 |
| *Laevo*-2,3-Butanediol | *Laevo*-2,3-Butanediol | 19132-06-0 | y=0.0077x+0.0052 | 0.9992 | 0.0499~49.99 |
| *Meso*-2,3-Butanediol | *Laevo*-2,3-Butanediol | 19132-06-0 | y=0.0077x+0.0052 | 0.9992 | 0.0499~49.99 |
| Hexanoic acid | Hexanoic acid | 142621 | y=0.4318x+0.0842 | 0.9986 | 0.0498~24.998 |
| Octanoic acid | Octanoic acid | 124072 | y =5.3255x+0.5776 | 0.9931 | 0.001~4.987 |
| Decanoic acid | Octanoic acid | 124072 | y =5.3255x+0.5776 | 0.9931 | 0.001~4.987 |
| Ethyl acetate | Ethyl acetate | 141786 | y=0.0235x-0.0056 | 0.9996 | 0.505~505.12 |
| 3-Methylbutyl acetate | 3-Methylbutyl acetate | 123922 | y=1.5142x+0.0144 | 0.9994 | 0.0499~2.499 |
| Phenethyl acetate | Phenethyl acetate | 103457 | y=4.6265x-0.4209 | 0.9985 | 0.0499~2.4926 |
| 2-Methylpropyl acetate | 2-Methylpropyl acetate | 110190 | y=0.4531x-0.0005 | 0.9994 | 0.05~2.508 |
| Ethyl propanoate | Ethyl propanoate | 105373 | y=0.1177x+0.0348 | 0.9998 | 0.2~20.0688 |
| Ethyl butanoate | Ethyl butanoate | 105544 | y=0.6483x-0.0002 | 0.9996 | 0.05~2.506 |
| Ethyl 2-methylpropanoate | Ethyl 2-methylpropanoate | 97621 | y=0.5475x-0.0085 | 0.995 | 0.02~0.99 |
| Ethyl hexanoate | Ethyl hexanoate | 123660 | y=6.1052x+0.1752 | 0.9976 | 0.025~2.52 |
| Ethyl 2-hexenoate | Ethyl hexanoate | 123660 | y=6.1052x+0.1752 | 0.9976 | 0.025~2.52 |
| Ethyl heptanoate | Ethyl heptanoate | 106309 | y=18.235x-0.2627 | 0.9975 | 0.0025~0.501 |
| Ethyl octanoate | Ethyl octanoate | 106321 | y=33.409x+0.1658 | 0.9962 | 0.05~1 |
| Ethyl decanoate | Ethyl decanoate | 110383 | y=55.592x-0.8634 | 0.9997 | 0.025~5.005 |
| Ethyl phenylacetate | Ethyl phenylacetate | 101973 | y=4.7848x+0.0047 | 0.9987 | 0.025~2.49 |
| Ethyl undecanoate | Ethyl dodecanoate | 106332 | y=56.636x-1.9287 | 0.999 | 0.02~1 |
| Ethyl dodecanoate | Ethyl dodecanoate | 106332 | y=56.636x-1.9287 | 0.999 | 0.02~1 |
| Ethyl tetradecanoate | Ethyl dodecanoate | 106332 | y=56.636x-1.9287 | 0.999 | 0.02~1 |
| Diethyl succinate | Ethyl hexanoate | 123660 | y=6.1052x+0.1752 | 0.9976 | 0.025~2.52 |
| Methyl decanoate | Methyl decanoate | 110429 | y=44.356x-2.3644 | 0.993 | 0.025~2.508 |
| 2-Methylpropyl decanoate | Ethyl dodecanoate | 106332 | y=56.636x-1.9287 | 0.999 | 0.02~1 |
| Octanoic acid, 3-methylbutyl ester | Ethyl dodecanoate | 106332 | y=56.636x-1.9287 | 0.999 | 0.02~1 |
| Ethyl 9-decenoate | Ethyl decanoate | 110383 | y=55.592x-0.8634 | 0.9997 | 0.025~5.005 |
| 3-Methylbutyl hexanoate | 3-Methylbutyl hexanoate | 2198610 | y = 30.26x-0.2062 | 0.9954 | 0.005~0.5011 |
| β-Damascenone | β-Damascenone | 23726912 | y=0.5861x-0.0013 | 0.9998 | 0.0055~0.55 |
| Benzaldehyde | Benzaldehyde | 100527 | y=2.9556x+0.021 | 0.998 | 0~0.4992 |
| 2-Nonanone | 2-Nonanone | 821556 | y =11.371x+0.002 | 0.9996 | 0.001~0.2 |
| 4-Heptanone, 2,6-dimethyl- | 4-Heptanone, 2,6-dimethyl- | 108838 | y =4.64x+0.5309 | 0.9989 | 0.005~0.501 |

All the analyses of the standard are purchased from Shanghai Aladdin Biochemical Technology Co., Ltd., for chromatographic purity pure. The concentration of these compounds is expressed as relative areas (to 4-methyl, 2-pentanol).

**Supplementary Table S4.** Odour activity values (OAVs) of main aroma compounds (OAV > 0.1) in different treatments wines at the end of alcoholic fermentation.

| Aroma compounds | Threshold (ug/L)^#^ | Aroma descriptor^#^ | SC | ZBI-2 | ZBI-3 | ZBII-2 | ZBII-3 | PKI-2 | PKI-3 | PKII-2 | PKII-3 |
| --- | --- | --- | --- | --- | --- | --- | --- | --- | --- | --- | --- |
| 1-Hexanol | 1100 | Herbaceous, grass, woody | 0.57 | 0.56 | 0.67 | 0.67 | 0.67 | 0.37 | 0.52 | 0.55 | 0.43 |
| (E)-3-Hexen-1-ol | 1000 | Herbaceous, green | 0.18 | 0.19 | 0.20 | 0.21 | 0.20 | 0.20 | 0.19 | 0.21 | 0.20 |
| (Z)-3-Hexen-1-ol | 1000 | Herbaceous, green, bitter, fatty | 0.20 | 0.19 | 0.20 | 0.20 | 0.19 | 0.18 | 0.19 | 0.19 | 0.19 |
| 2-Methyl-1-propanol | 75000 | Alcohol, solvent, green, bitter | 0.63 | 0.73 | 1.08 | 0.86 | 0.89 | 1.95 | 2.03 | 1.65 | 1.79 |
| 3-Methyl-1-butanol | 60000 | Solvent, sweet, alcohol, nail polish | 4.51 | 2.70 | 2.85 | 3.85 | 2.57 | 3.26 | 3.10 | 3.67 | 3.59 |
| 3-Methyl-1-pentanol | 500 | Pungent, solvent, green | 1.01 | 0.48 | 0.42 | 0.63 | 0.45 | 0.41 | 0.41 | 0.43 | 0.43 |
| 1-Heptanol | 200 | Oily | 0.58 | 0.08 | - | 0.15 | - | - | - | - | - |
| Phenylethyl alcohol | 14000 | Roses, hone | 17.74 | 4.08 | 2.86 | 11.26 | 3.70 | 3.79 | 3.62 | 4.78 | 4.28 |
| *Leavo*-2,3-Butanediol | 150000 | Fruity, sweet, butter | 0.30 | 0.17 | 0.23 | 0.24 | 0.21 | 0.30 | 0.16 | 0.18 | 0.17 |
| *Meso*-2,3-Butanediol | 150000 | Fruity, sweet, butter | 0.11 | 0.05 | 0.09 | 0.06 | 0.07 | 0.17 | 0.18 | 0.20 | 0.21 |
| Hexanoic acid | 420 | Cheese, fatty | 1.76 | 0.44 | 0.28 | 0.56 | 0.33 | - | - | - | - |
| Octanoic acid | 500 | Rancid, cheese, fatty acid | 0.73 | 0.10 | 0.03 | 0.20 | 0.09 | - | - | 0.05 | 0.03 |
| Ethyl acetate | 7500 | Pineapple, fruity, solvent | 6.26 | 8.97 | 20.78 | 6.52 | 9.64 | 33.07 | 36.11 | 42.47 | 49.23 |
| 3-Methylbutyl acetate | 160 | Banana | 1.84 | 2.03 | 2.28 | 1.72 | 1.91 | 7.38 | 9.21 | 9.63 | 13.51 |
| Phenethyl acetate | 250 | Rose, honey, tobacco | 0.62 | 0.44 | 0.48 | 0.58 | 0.50 | 0.51 | 0.55 | 0.65 | 0.68 |
| 2-Methylpropyl acetate | 1600 | Strawberry, fruity, flowery | 0.04 | 0.05 | 0.06 | 0.04 | 0.04 | 0.16 | 0.17 | 0.15 | 0.19 |
| Ethyl propanoate | 1800 | Sweet, fruity | 0.12 | 0.15 | 0.31 | 0.18 | 0.17 | 0.38 | 0.39 | 0.34 | 0.38 |
| Ethyl butanoate | 400 | Banana, pineapple, strawberry | 0.43 | 0.49 | 0.46 | 0.42 | 0.48 | 0.35 | 0.28 | 0.27 | 0.26 |
| Ethyl 2-methylpropanoate | 15 | Sweet, fruity | 2.61 | 2.84 | 3.87 | 2.76 | 2.89 | 8.90 | 9.94 | 10.77 | 13.34 |
| Ethyl hexanoate | 80 | Banana, green apple | 4.62 | 1.82 | 0.52 | 2.01 | 0.97 | 0.92 | 0.77 | 1.05 | 0.50 |
| Ethyl octanoate | 580 | Sweet, floral, fruity, banana, pear | 1.29 | 0.35 | 0.11 | 0.38 | 0.21 | 0.28 | 0.13 | 0.25 | 0.13 |
| Ethyl decanoate | 200 | Fruity, fatty, pleasant | 2.84 | 0.54 | 0.17 | 0.87 | 0.37 | 0.28 | 0.16 | 0.31 | 0.17 |
| Methyl decanoate | 1.2 | nf | 45.20 | - | 44.59 | 44.62 | 44.58 | - | - | - | - |
| Ethyl 9-decenoate | 100 | Rose | 0.69 | 0.25 | 0.21 | 0.26 | 0.22 | 0.22 | 0.21 | 0.22 | 0.21 |
| *β*-Damascenone | 0.14 | Sweet, exotic flowers, stewed apple | 111.11 | 71.82 | 71.38 | 81.05 | 79.35 | 66.30 | 64.39 | 69.73 | 71.34 |
| 2-Nonanone | 1.6 | Fruity | 0.83 | 0.32 | 0.26 | 0.21 | 0.31 | 0.35 | 0.00 | 0.00 | 0.08 |

SC: single inoculation with *S. cerevisiae* EC1118; ZBI-2: sequential inoculation with *Z. bailii* BJVI11007, followed by inoculation with EC1118 after 2 days; ZBI-3: sequential inoculation with *Z. bailii* BJVI11007, followed by inoculation with EC1118 after 3 days; ZBII-2: sequential inoculation with *Z. bailii* BJII45005, followed by inoculation with EC1118 after 2 days; ZBII-3: sequential inoculation with *Z. bailii* BJII45005, followed by inoculation with EC1118 after 3 days; PKI-2: sequential inoculation with *P. kudriavzevii* BJIV53006, followed by inoculation with EC1118 after 2 days; PKI-3, sequential inoculation with *P. kudriavzevii* BJIV53006, followed by inoculation with EC1118 after 3 days; PKII-2, sequential inoculation with *P. kudriavzevii* BJII44006, followed by inoculation with EC1118 after 2 days; PKII-3: sequential inoculation with *P. kudriavzevii* BJII44006, followed by inoculation with EC1118 after 3 days.

- means the compound was not detected by GC–MS in the corresponding wine sample.

nf means not found aroma descriptor.

^#^ Reference of threshold and aroma descriptor: Cai, J., Zhu, B. Q., Wang, Y. H., Lu, L., Lan, Y. B., Reeves, M. J., Duan, C. Q. (2014). Influence of pre-fermentation cold maceration treatment on aroma compounds of *Cabernet Sauvignon* wines fermented in different industrial scale fermenters. *Food Chem*. 154, 217–229. doi: 10.1016/j.foodchem.2014.01.003; Peng, C., Wen, Y., Tao, Y., Lan, Y. (2013). Modulating the formation of Meili wine aroma by pre-fermentative freezing process. *J. Agric. Food Chem.* 61, 1542-1553. doi: 10.1021/jf3043874; Tao, Y., Zhang, L. (2010). Intensity prediction of typical aroma characters of *Cabernet Sauvignon* wine in Changli County (China). *LWT- Food Sci. Technol.* 43, 1550-1556. doi: 10.1016/j.lwt.2010.06.003; Welke, J., Zanus, M., Lazzarotto, M., Zini, C. (2014). Quantitative analysis of headspace volatile compounds using comprehensive two-dimensional gas chromatography and their contribution to the aroma of chardonnay wine. *Food Res. Int.* 59(1), 85-99. doi: 10.1016/j.foodres.2014.02.00

# Supplementary Figure


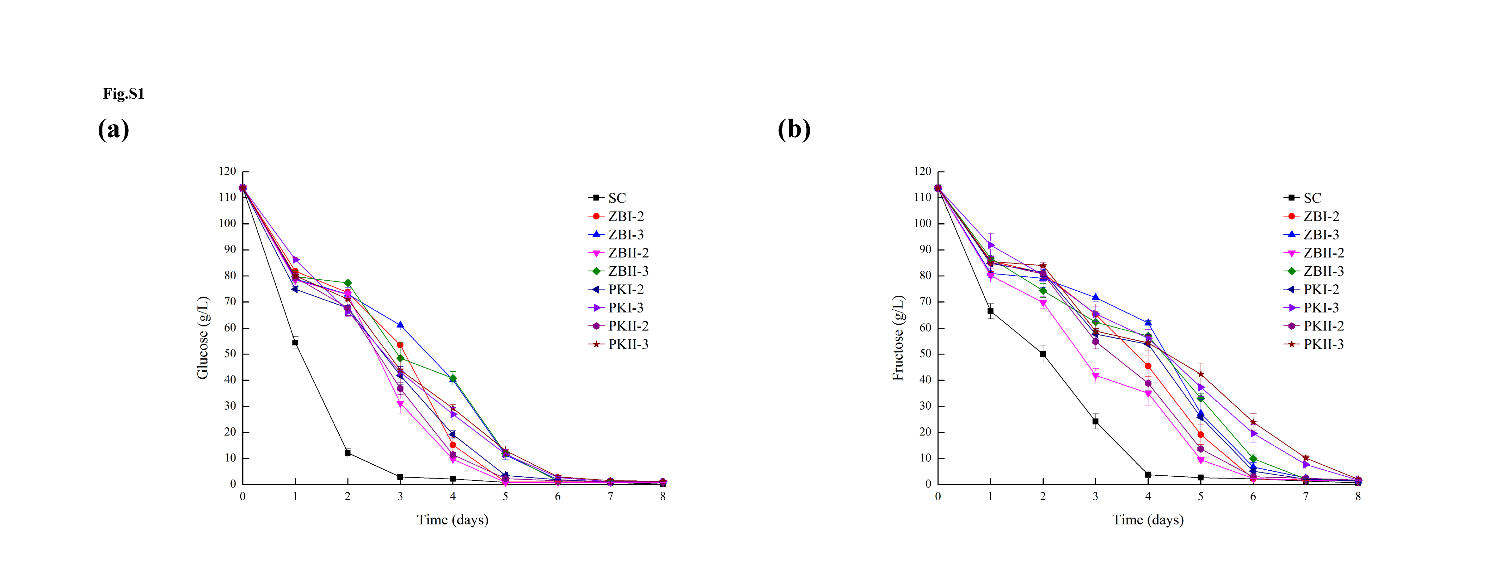


**Supplementary Figure 1.** Evolution of glucose (a) and fructose (b) during alcoholic fermentation. SC: single inoculation with *S. cerevisiae* EC1118; ZBI-2: sequential inoculation with *Z. bailii* BJVI11007, followed by inoculation with EC1118 after 2 days; ZBI-3: sequential inoculation with *Z. bailii* BJVI11007, followed by inoculation with EC1118 after 3 days; ZBII-2: sequential inoculation with *Z. bailii* BJII45005, followed by inoculation with EC1118 after 2 days; ZBII-3: sequential inoculation with *Z. bailii* BJII45005, followed by inoculation with EC1118 after 3 days; PKI-2: sequential inoculation with *P. kudriavzevii* BJIV53006, followed by inoculation with EC1118 after 2 days; PKI-3, sequential inoculation with *P. kudriavzevii* BJIV53006, followed by inoculation with EC1118 after 3 days; PKII-2, sequential inoculation with *P. kudriavzevii* BJII44006, followed by inoculation with EC1118 after 2 days; PKII-3: sequential inoculation with *P. kudriavzevii* BJII44006, followed by inoculation with EC1118 after 3 days.
